# The State- and Trait-Level Effects and Candidate Mechanisms of Four Mindfulness-Based Cognitive Therapy (MBCT) Practices: Two Exploratory Studies

**DOI:** 10.1007/s12671-023-02193-6

**Published:** 2023-08-24

**Authors:** Shannon Maloney, Christina Surawy, Maryanne Martin, Jesus Montero-Marin, Willem Kuyken

**Affiliations:** 1 Department of Psychiatry, University of Oxford, Oxford, OX37JX UK; 2 Department of Experimental Psychology, University of Oxford, Anna Watts Building, Woodstock Road, Oxford, OX2 6GG UK; 3 Teaching, Research & Innovation Unit, Parc Sanitari Sant Joan de Déu, Sant Boi de Llobregat, Spain; 4 Consortium for Biomedical Research in Epidemiology & Public Health (CIBER Epidemiology and Public Health - CIBERESP), 28029 Madrid, Spain

**Keywords:** Mindfulness-based cognitive therapy, MBCT, Mindfulness practice, Mechanisms

## Abstract

**Objectives:**

The primary aim was to explore state- and trait-level effects and candidate mechanisms of four Mindfulness-Based Cognitive Therapy (MBCT) practices.

**Method:**

One hundred sixty adults self-selected from the general population were randomized to one of four mindfulness practices: body scan, mindful movement, breath and body, and befriending. Study 1 explored state-level self-compassion, mindfulness, decentering (mechanisms), and pleasantness of thoughts, emotions, and body sensations at multiple time points using two single mindfulness sessions. Study 2 explored trait-level self-compassion, mindfulness, decentering, interoceptive awareness, attentional control (mechanisms), anxiety, depression, and psychological quality of life pre-post 2 weeks of daily practice.

**Results:**

In study 1, state-level effects were demonstrated in all candidate mechanisms and outcomes within the whole sample across time points (*d* = 0.27 to 0.86), except for state decentering. After controlling for pre-scores and additional covariates, no between-group effects were found (*p* = 0.050 to 0.973). In study 2, trait-level effects were demonstrated in psychological quality of life and most candidate mechanisms within the whole sample (*d* = 0.26 to 0.64) but no between-group effects were found (*p* = 0.080 to 0.805). Within the whole sample, after controlling for pre-scores, changes in mindfulness, self-compassion, decentering, and interoceptive awareness (i.e. body listening) were associated with improvements in psychological quality of life (*r* = 0.23 to 0.40) and self-led mindfulness practice (*r* = 0.18 to 0.23).

**Conclusions:**

Future research should test the generated hypotheses using well-designed, adequately powered, and theory-driven studies that address universal and specific mechanisms in different populations and contexts.

**Pre-registration:**

This study is not pre-registered.

**Supplementary Information:**

The online version contains supplementary material available at 10.1007/s12671-023-02193-6.

Mindfulness-Based Cognitive Therapy (MBCT) was first developed in the early 2000s for individuals with recurrent depression (Segal et al., 2018). Since then, it has been adapted for new populations and contexts, including for the general population through the “Finding Peace in a Frantic World” programme (M-FP; Williams & Penman, 2011) and the more intensive “MBCT for Life” course (Strauss et al., 2021). MBCT typically constitutes an 8-week curriculum comprised of weekly group-based sessions led by a trained mindfulness teacher. Recent meta-analyses have provided support for MBCT as an efficacious treatment strategy for relapse prevention compared to no intervention (Kuyken et al., 2016) and there is evidence that suggests that MBCT may be comparable to some active treatments (e.g., treatment-as-usual and anti-depressant medication) (Biesheuvel-Leliefeld et al., 2015; McCartney et al., 2021). There is also a growing body of evidence that has demonstrated preliminary efficacy of MBCT adaptations in general population samples (e.g., secondary school teachers, university students, healthcare workers, and workplace employees) (Beshai et al., 2016; de Bruin et al., 2020; Medlicott et al., 2021; Montero-Marin et al., 2021; Strauss et al., 2021; Taylor et al., 2014). A recent meta-analysis found that, compared to no intervention, mindfulness-based programmes (MBPs) in non-clinical settings promoted improvements in mental health and well-being with small to medium effects (Galante et al., 2021). However, this area of research is still in its early stages (Creswell, 2017; Dimidjian & Segal, 2015; Galante et al., 2021; Van Dam et al., 2018) with generally low to moderate quality trials that lack a consensus on a range of factors, such as the programme protocol (e.g., optimal dosage) and types of active comparators. Moreover, there is limited understanding of how generalizable effects are across the entire population.

Mindfulness can be described as a multidimensional construct that refers to a range of processes and practices (Feldman & Kuyken, 2019; Van Dam et al., 2018). Theoretical models (Feldman & Kuyken, 2019; Segal et al., 2018; Shapiro et al., 2006) argue that mindfulness involves bringing attention and awareness intentionally to the present-moment experience (e.g., thoughts, emotions, bodily sensations, and behaviours) with attitudes such as acceptance, compassion, and curiosity. In the process of increasing one’s attention and awareness, along with these attitudes of mindfulness, this then allows the individual to take a wider perspective (decentering or meta-cognitive awareness) which then leads to greater discernment to make an empowered choice on how to respond to both internal and external stimuli. Systematic reviews and meta-analyses have provided support for the central concept of mindfulness along with overlapping yet distinct processes (e.g., self-compassion and decentering) as candidate mechanisms underlying MBCT and other MBPs (Alsubaie et al., 2017; Gu et al., 2015; Maddock & Blair, 2023; van der Velden et al., 2015). Processes relating to attention and awareness (e.g., attentional control and interoceptive awareness) have also been examined as mechanisms of change (Chambers et al., 2008; Chiesa et al., 2011; Hanley et al., 2017), but warrant further investigation. In specific populations (i.e. recurrent depression), mechanisms of decentering, rumination, and cognitive reactivity seem to be integral to the process of change (Bieling et al., 2012; Cladder-Micus et al., 2018; Fissler et al., 2016; Segal et al., 2019; van Aalderen et al., 2012). Additionally, in general population samples, there is preliminary support for mindfulness and self-compassion as candidate mechanisms (Medlicott et al., 2021; Montero-Marin et al., 2021; Strauss et al., 2021) that may help shift a wider distribution of the population more towards flourishing and away from languishing. However, the field has not reached a consensus on the mechanisms that are specific to and shared across different MBCT adaptations and populations (Goldberg, 2022). There is currently work underway (Maloney et al., submitted), which aims to unpack the mechanisms of MBPs in different population samples across mental health and well-being outcomes. However, more experimental work is required to add to this growing literature and, in parallel, more work is needed to clarify the operational definitions of the candidate mechanisms and outcomes in relation to the theory of change and the intention behind the MBP (e.g., treatment, prevention, promotion).

Kazdin’s (2007) seminal paper provides a framework for establishing a mechanism of change and it proposes specific design and data-analytic approaches. Beyond RCTs, Kazdin suggests that component analyses can also help uncover a mechanism of change. These approaches (e.g., dismantling trials) consider a programme as a “package” of components that arguably have an independent impact on outcome. In the context of MBPs, there are MBP-specific (e.g., mindfulness practices) and non-specific (e.g., cognitive-behavioural techniques, group influence, and teacher support) elements (Goldberg, 2022). The intention behind these analyses is to first identify components and to then understand why they produce independent change (Kazdin, 2007). Mindfulness practices are regarded as a crucial ingredient of MBPs. Crane et al. (2017) use a metaphor from weaving to define an MBP and describe the universal elements as the “warp” and the specific elements as the “weft”. In their definition, mindfulness practices are indicated as a universal element (“the warp”) that is shared across all MBPs. Early research has provided empirical support (Blanck et al., 2018; Carmody & Baer, 2008; Sauer-Zavala et al., 2013; Schumer et al., 2018; Singer & Kok, 2017) demonstrating that mindfulness practices can produce independent effects outside the framework of MBPs. Following Kazdin’s (2007) recommendations, the next step involves investigating why (i.e. through which mechanisms) these individual mindfulness practices produce these effects in which some preliminary work has already been conducted. However, this area of work is currently underdeveloped and the available studies fail to address many key requirements in establishing a mechanism of change (Kazdin, 2007).

In the context of a burgeoning area of research, the aim of the current study was to build on the evidence for candidate mechanisms of MBCT specifically in the context of general population samples. Following Kazdin’s (2007) framework, the current paper explored mechanisms grounded in theory and evidence and utilized a dismantling approach to understand the mechanisms of individual mindfulness practices, as one core component, to further understand how and why MBCT works. Ultimately, a deeper understanding of how and why components of MBCT work in isolation will help identify active ingredients that can be optimized and more thoughtfully considered when adapting MBCT to different population samples and contexts.

Logic diagrams are often used to help visualize and describe the theoretical framework of how and why (through which mechanisms) one may expect a programme, or in this case one core component of a programme, produces change in outcome (Moore et al., 2015). In the current study, the potential outcomes and mechanisms of four individual mindfulness practices, from an MBCT programme adapted for a general population sample (M-FP; Williams & Penman, 2011), are explored. The four MBCT practices investigated in the current study include the breath and body, body scan, mindful movement, and befriending practice (Supplement A in the Supplementary Material). In the context of the M-FP programme, the breath and body practice is introduced in the first week of the 8-week curriculum. This practice involves bringing one’s attention to the breath with the aim of stabilizing attention and recognizing automatic pilot, which has been described as the occurrence of automatically paying attention elsewhere and missing what is happening in the present moment. The body scan is introduced in the second week and involves bringing attention to different parts of the body in turn, with the aim of reintegrating the mind and body, and seeing the mind’s reactivity. The mindful movement practice is introduced in the third week and involves simple stretches and reminders of not pushing the body beyond its limits, with the aim of helping individuals anchor awareness in the moving body and become aware of striving. The befriending practice is introduced in the sixth week and involves imagining different individuals (e.g., a loved one, a stranger, a neutral person) and wishing them all well with the aim of cultivating kindness and letting go of the past and a sense of permanency (Williams & Penman, 2011). A trained mindfulness teacher was consulted when deciding which practices from the M-FP programme to explore. It was recommended to not explore the sounds and thoughts and experiencing difficulty practices, in the context of the proposed study design, given that these practices are generally practiced in combination with another practice (e.g., breath and body) to help individuals settle before engaging with more complex instructions.

Outcomes relating to mental languishing and flourishing are explored to map onto the intentions behind the M-FP programme. As a mental health promotion strategy, the aim behind the M-FP programme is to help any adult interested in cultivating a mindfulness practice experience positive mental health (e.g., greater quality of life) and find ways of breaking cycles of anxiety and depression to prevent further deterioration. Candidate mechanisms (mindfulness, self-compassion, decentering, attentional control, and interoceptive awareness) grounded in theory (Feldman & Kuyken, 2019; Segal et al., 2018; Shapiro et al., 2006) and existing evidence (Alsubaie et al., 2017; Chambers et al., 2008; Chiesa et al., 2011; Gu et al., 2015; Hanley et al., 2017; Maddock & Blair, 2023; van der Velden et al., 2015) are explored. Mindfulness has been conceptualized both as a state, which can be tapped into whilst practicing, and also as a trait which is more stable and related to cultivating mindfulness in your everyday life (Kiken et al., 2015; Lau et al., 2006; Medvedev et al., 2017). In the current study, potential state-level and trait-level changes in candidate mechanisms in relation to relevant state-level and trait-level outcomes were investigated. For example, state-level outcomes (i.e. changes in pleasantness of emotions, thoughts, and body sensations) were chosen based on their relevance to the proposed trait-level outcomes (i.e. depression and anxiety symptoms and psychological quality of life) and state-level mechanisms (i.e. state mindfulness, decentering, and self-compassion) were chosen based on their relevance to the proposed trait-level mechanisms (i.e. trait mindfulness, decentering, and self-compassion). Overall, there is limited research on the state-level versus trait-level changes of candidate mechanisms (Carmody et al., 2008; Kiken et al., 2015), especially in the context of individual mindfulness practices (i.e. as a key component of MBCT). Moreover, in light of research that has found a relationship between the amount of self-led mindfulness practice completed during the intervention (Parsons et al., 2017) and previous levels of mindfulness experience (Thompson & Waltz, 2007) with outcome, the current paper also explored the influence of these variables on changes in the candidate mechanisms and outcomes. Please see Supplement B in the Supplementary Material for the logic diagram, which provides a simplified visual of the key components (MBCT practices, candidate mechanisms, and outcomes) explored in the current study.

Following Kazdin’s (2007) framework, the current paper first explored the effects of four MBCT practices on potential state-level and trait-level mechanisms and outcomes. Key requirements in establishing a mechanism of change were also partially explored by examining (1) the relationship between change in the candidate mechanism and change in outcome (strong association criterion) and (2) the extent to which a larger dose (e.g., more self-led mindfulness practice) relates to change in candidate mechanisms and outcomes (gradient criterion). The current paper includes one participant sample to explore state- and trait-level effects and potential mechanisms of change. Different aspects of the study design (Fig. 2) are used to explore state- versus trait-level variables, and therefore, the paper has been broken down into two separate parts (Study 1 and Study 2). In an effort to explore state-level changes, Study 1 explored changes in proposed outcomes (pleasantness of thoughts, emotions, and body sensations) and candidate mechanisms (state mindfulness, self-compassion, and decentering) across multiple time points using single one-off mindfulness sessions. These state-level outcomes and mechanisms were explored across two time points between a 2-week intervention period of daily mindfulness practice to explore the extent to which increases in state-level change occur over time, which has been supported in the literature (Kiken et al., 2015). Study 2 explored trait-level changes in proposed outcomes (psychological quality of life, anxiety, and depression) and mechanisms (trait mindfulness, self-compassion, decentering, attentional control, and interoceptive awareness) before and after the same 2-week intervention period used in Study 1. The 2-week intervention period was chosen based on past research that has demonstrated that this dosage of mindfulness can promote change in outcomes (Schumer et al., 2018). Ultimately, the aim of the current paper is to generate hypotheses regarding the state-level and trait-level effects and mechanisms of four unique MBCT practices for future research to test in a larger sample.

## Study 1

### Method

#### Participants

**Fig. 1:**
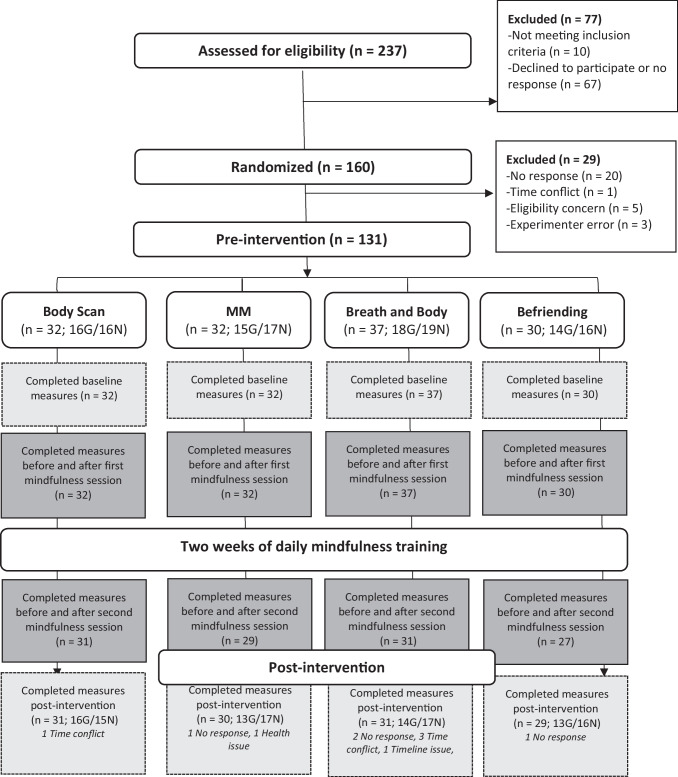
Flow diagram. The figure depicts the CONSORT flow diagram from screening for eligibility to pre-intervention to post-intervention. **Key**: MM = Mindful Movement; G = MBCT Graduate; N = MBP Naïve; Dark grey boxes = Study 1; Light grey boxes with dotted line = Study 2

One hundred and sixty self-selected adults who presented no clinical symptoms for depression and anxiety were recruited and randomized in total. Out of the 160 adults, 18.13% (*n* = 29) did not provide data at baseline, either due to no response (*n* = 20), a time conflict (*n* = 1), an eligibility concern that was identified later (*n* = 5), or experimenter error (*n* = 3), leaving 131 participants subsequently included (Fig. 1). At baseline, there were no significant differences across the four experimental groups in terms of symptoms of depression and anxiety, psychological quality of life, gender, age, and previous levels of mindfulness experience (Table 1).

**Table 1:** Descriptive statistics of whole sample by experimental group at baseline

	Whole sample (*n* = 131)	Body scan (*n* = 32)	MM (*n* = 32)	Breath and body (*n* = 37)	Befriending (*n* = 30)	*p*
Gender, female (frequency)	99	26	24	30	19	0.418
%	75.60	81.30	75.00	81.10	63.30	
Age, *M* (*SD*)	41.46 (15.20)	39.03 (14.34)	41.78 (15.17)	42.49 (14.50)	42.43 (17.32)	0.749
Range	18–75	21–65	21–75	18–68	21–74	
Mindfulness experience, MBCT naïve (frequency)	68	16	17	19	16	0.995
%	51.90	50.00	53.10	51.40	53.30	
Depression, *M* (*SD*)	3.18 (2.98)	2.72 (2.65)	2.84 (2.84)	3.73 (3.41)	3.37 (2.92)	0.568
Range	0.00–14.00	0.00–9.00	0.00–11.00	0.00–14.00	0.00–10.00	
Anxiety, *M* (*SD*)	3.69 (3.23)	3.19 (2.92)	2.90 (3.03)	4.22 (3.64)	4.41 (3.09)	0.108
Range	0.00–14.00	0.00–10.00	0.00–14.00	0.00–14.00	0.00–12.00	
Psych QOL, *M* (*SD*)	65.11 (12.98)	64.32 (12.29)	67.71 (11.55)	62.16 (16.00)	66.81 (10.52)	0.286
Range	25.00–100.00	33.33–87.50	37.50–87.50	25.00–100.00	41.67–87.50	
Mindfulness, *M* (*SD*)	40.27 (6.38)	39.44 (5.46)	41.53 (6.17)	38.78 (7.16)	41.67 (6.22)	0.149
Range	25.00–57.00	31.00–52.00	30.00–53.00	25.00–57.00	32.00–54.00	

The table includes descriptive data of the sample at baseline using a complete-cases approach. Means (standard deviations), or frequencies (percentages) of baseline characteristics by whole sample and by mindfulness practice (body scan, mindful movement, breath and body, befriending) are reported. The one-way analysis of variance for non-parametric tests (Kruskal-Wallis) or the corresponding Fisher-Freeman-Halton Exact test was used to examine possible between-group differences at baseline. Key: MM = Mindful Movement. Depression was measured using the PHQ-9 (scores range from 0 to 27 with symptom severity cutoffs at 5 (mild), 10 (moderate), 15 (moderately severe), and 20 (severe)). Anxiety was measured using the GAD-7 (scores range from 0 to 21 with symptom severity cutoffs at 5 (mild), 10 (moderate), 15 (severe)). Psychological quality of life was measured using the psychological domain of the WHOQOL-BREF (scores range from 0 to 100; scores of 60 and above are interpreted as optimal). Mindfulness was measured using the FFMQ-15 (scores range from 12 to 60 (with the observing scale omitted) and the total score was calculated without the Observing subscale; the total score with the Observing subscale included was 50.26 (SD = 7.62) (scale range from 15 to 75) within the whole sample at baseline; in a selected sample of secondary school teachers, the average score was 51.5 (SD = 6.8) using all five subscales for the total score (Montero-Marin et al., 2021); in a selected sample of mostly individuals who have completed a formal mindfulness-based programme, the average score was 52.66 (SD = 9.14) using only four subscales (omitting the Observing subscale)) (Williams et al., 2022). For those that identified as MBCT naïve within the whole sample (*n* = 68), 55.9% of them reported that they had some other mindfulness experience (e.g., digital platforms such as Headspace or Calm)

The inclusion criteria were as follows: (1) aged 18 and above and (2) English-speaking. Participants who had completed an MBCT course, e.g., “MBCT for Depression” (Segal et al., 2018), “MBCT - Finding Peace in a Frantic World” (M-FP; Williams & Penman, 2011), and “MBCT for Life” (Strauss et al., 2021) were eligible to take part. The exclusion criteria were as follows: (1) scores of 15 or greater on PHQ-9 (moderately severe to severe symptoms) or GAD-7 (severe symptoms) or (2) presence of suicidal thoughts (score of 2 or 3 on item 9 of PHQ-9). The rationale for this exclusion criteria was to minimize potential harm (Baer et al., 2021), in light of the limited evidence for the effects of individual and unsupervised MBCT practices on mental health outcomes.

The current paper recruited individuals with a range of mindfulness experience, including those who have already completed an MBCT programme and who have not completed a formal MBP (e.g., MBCT or Mindfulness-Based Stress Reduction (MBSR)) (Crane et al., 2017). Out of those included at baseline (*n* = 131), 63 completed an MBCT programme prior to the start of the study (“MBCT graduates”) and the remaining 68 did not complete any formal MBP (“MBP naïve”). At baseline, we expected differences across these groups in terms of their trait mindfulness scores. The MBCT graduate group reported slightly higher trait mindfulness scores (*M* = 41.17, *SD* = 6.57) compared to the naïve group (*M* = 39.44, *SD* = 6.12) with small to medium effects; however, this difference was non-significant (*p* = 0.147) (Supplement C in the Supplementary Material). These groups may not be as distinct, in terms of their trait mindfulness scores, since the majority of the naïve group (55.9%) reported that they had some other form of mindfulness experience prior to the start of the study (e.g., through digital platforms such as Headspace or Calm). In light of these findings and the small sample size, no sub-group analyses were conducted. However, sensitivity analyses were undertaken whereby this variable along with other variables (i.e. perceived impact of the Covid-19 pandemic and format (online versus in-person)) was controlled for as covariates (Supplement E2 and F2 in the Supplementary Material).

#### Procedure

**Fig. 2:**
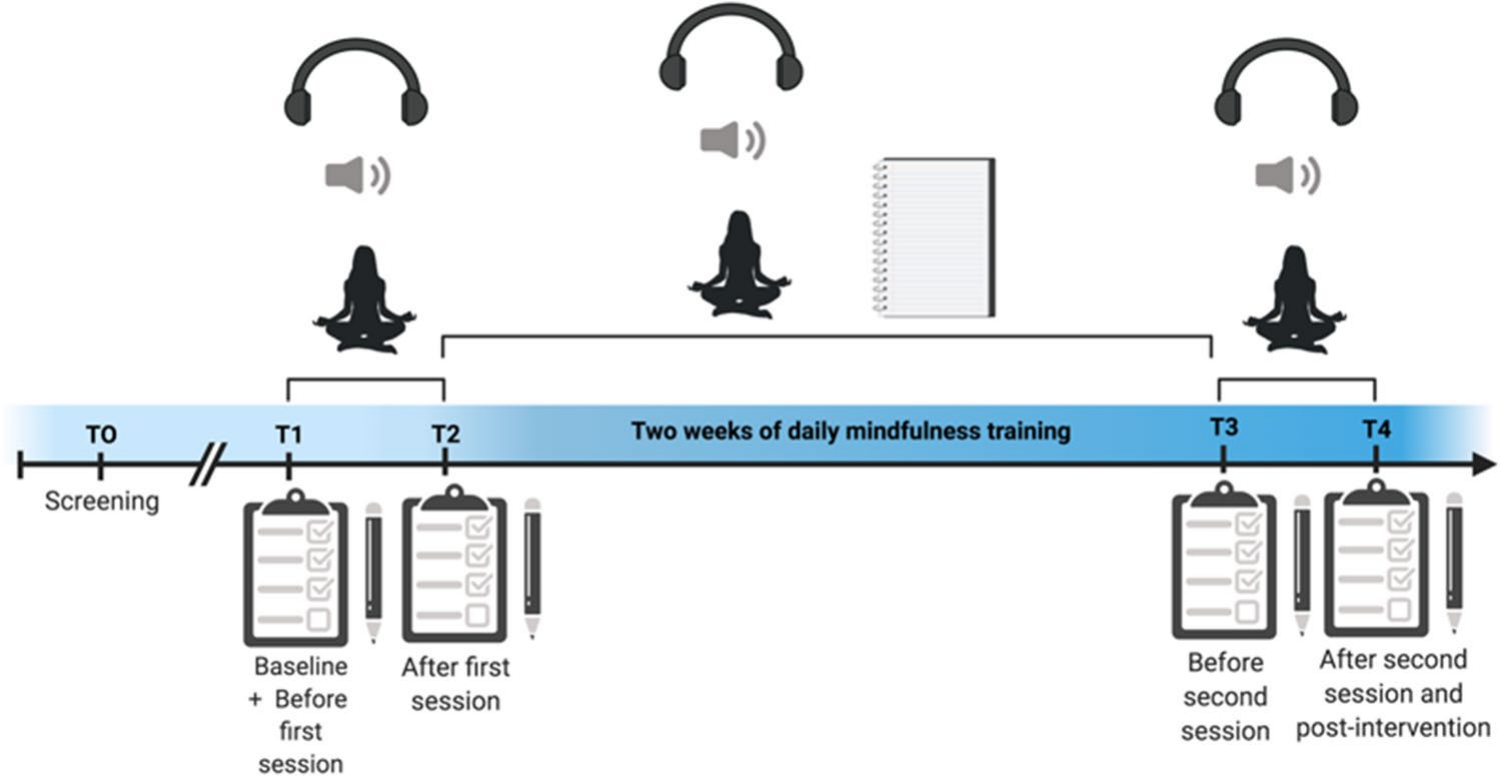
Survey administration. The figure outlines the survey administration from T0 (screening) to T1 (baseline and before the first mindfulness session) to T2 (after the first mindfulness session) to T3 (before the second mindfulness session) to T4 (after the second mindfulness session and post-intervention). Between T2 and T3, participants engage in 2 weeks of daily mindfulness practice and log their daily mindfulness practice using a journal log. Surveys were administered at **T0** (GAD-7, PHQ-9), **T1** (WHOQOL-BREF, SCS-SF, EQ, ACS-S, FFMQ-SF, MAIA-2, VAS for body sensations, emotions, and thoughts, and SSC), **T2** (TMS, SMS, and VAS for body sensations, emotions, and thoughts, and SSC), **T3** (VAS for body sensations, emotions, and thoughts and SSC), and **T4** (GAD-7, PHQ-9, WHOQOL-BREF, SCS-SF, EQ, ACS-S, FFMQ-SF, MAIA-2, TMS, SMS, and VAS for body sensations, emotions and thoughts, and SSC). **Key:** GAD-7 = Generalized Anxiety Disorder (GAD-7); PHQ-9 = Patient Health Questionnaire (PHQ-9); Mindfulness level = MBCT Graduate or MBP Naïve; WHOQOL-BREF = World Health Organization Quality of Life-BREF questionnaire (WHOQOL-BREF); SCS-SF = Self-Compassion Scale Short Form (SCS-SF); EQ = Experiences Questionnaire (EQ); ACS-S = Attentional Control Scale (ACS-S); FFMQ-15 = Five Facet Mindfulness Questionnaire (FFMQ-15); MAIA-2 = Multidimensional Assessment of Interoceptive Awareness Version 2 (MAIA-2); VAS = visual analogue scales; SSC = State self-compassion VAS. The figure was adapted from template developed by BioRender.com (2022). Retrieved from https://app.biorender.com/biorender-templates

Participants were recruited on a rolling basis through the University of Oxford departmental and college websites, newsletters, and social media platforms. Participants were first screened for eligibility at T0 (Time point 0; Fig. 2). A researcher outside of the core research team created a computer-generated randomization list using blocked stratified randomization with random block sizes of eight (to minimize expectation biases for the experimenter) and strata formed by previous mindfulness experience (MBCT graduate or MBP naïve). Participants were randomly assigned to one of four experimental groups: body scan, mindful movement, breath and body, and befriending practice. The researcher who collected the data was informed of the allocation the day of each individual session and participants were blind to the name of their allocated practice to help minimize expectation biases. Participants were invited to a lab session where they completed a battery of questionnaires before and after one single mindfulness session using audio from the M-FP CD guidance (Williams & Penman, 2011) (Time point 1 to Time point 2; T1–T2). For 2 weeks, participants then engaged with one mindfulness practice daily at home and tracked adherence using a journal log (Time point 2 to Time point 3; T2–T3). After 2 weeks, participants were invited to a second lab session where they completed a battery of questionnaires before and after one single mindfulness session (Time point 3 to Time point 4; T3–T4). Participants were offered only one type of mindfulness practice for the full duration of the study period, depending on their random allocation. Study 1 included data collected before and after the first and second single mindfulness sessions (Time points T1–T2, T3–T4, and T2–T4). Efforts were made to minimize differences beyond the core guidance (e.g., length of practice and mindfulness teacher) across the four experimental groups. Therefore, the lengths of silences were either shortened or lengthened, without removing them entirely or making any changes to the script, so that each guidance ranged from 10 to 12 min. The same mindfulness teacher was also used for each audio recording.

#### Measures

All measures for Study 1 were administered before and after the single mindfulness sessions (T1–T2 and T3–T4), except for the state decentering and mindfulness measures which were administered only after each mindfulness session (T2, T4) to collect retrospective data on their mindfulness experience during a specific timeframe (e.g., in the last 10–12 min) as instructed.

Pleasantness of thoughts, emotions, and body sensations were considered as state-level outcomes and were measured using a single-item visual analogue scale (VAS) for each content (three in total) at T1, T2, T3, and T4. These items were adapted from previous work (Smallwood et al., 2016; van der Velden et al., 2023). Item example: “On a scale of 0-100, the body sensations that I am experiencing are unpleasant (0), to neutral (50), to pleasant (100).” Internal consistency values were not obtained in the present study due to single-item VAS (Wewers & Lowe, 1990).

State self-compassion, decentering, and mindfulness were explored as candidate state-level mechanisms. In the current study, changes in state mindfulness were associated with changes in state decentering and self-compassion (*r* = 0.34 to 0.45, *p* < 0.001). Self-compassion generally involves the ability to bring kindness and acceptance towards the human condition and suffering (Neff, 2003) and was measured using a two-item VAS (Kirschner et al., 2019) adapted from the Self-Compassion Scale – Short Form (SCS-SF; Raes et al., 2011) at T1, T2, T3, and T4. These two items were positively correlated at baseline (T1: *r*_(129)_ = 0.73, *p* < 0.001) and were subsequently combined to create a composite score. The two items included the following: “On a scale to 0-100, I feel like not being kind and understanding towards myself at all (0) to feeling like being very kind and understanding towards myself (100)” and “On a scale of 0-100, I am not tolerant of my flaws and inadequacies at all (0) to I am very tolerant of my flaws and inadequacies (100).” The internal consistency values for the two-item VAS have demonstrated acceptable reliability (*α* = 0.73) in a sample of university students (Kirschner et al., 2019). In the current paper, the internal consistency values were *α* = 0.85 (T1), *α* = 0.87 (T2), *α* = 0.65 (T3), and *α* = 0.80 (T4).

State decentering has been defined as the ability to take a wider perspective of one’s current experience without getting carried away by thoughts and feelings in the present moment (Teasdale et al., 2002) and was measured using the 7-item Decentering subscale of the Toronto Mindfulness Scale (TMS; Teasdale et al., 2002) at T2 and T4. Items such as “I experienced myself as separate from my changing thoughts and feelings” were rated on a 5-point Likert scale (from 0 = *Not at all* to 4 = *Very much*). The TMS Decentering subscale has demonstrated good reliability (*α* = 0.84) in a sample of participants with a range of mindfulness experience (Lau et al., 2006). In the current paper, the internal consistency values were *α* = 0.83 for T2 and *α* = 0.84 for T4.

State mindfulness has been defined as present-moment awareness characterized by attitudes of curiosity, openness, and acceptance (Tanay & Bernstein, 2013) and was measured using the 21-item “State Mindfulness Scale” (SMS; Tanay & Bernstein, 2013) at T2 and T4. Items such as “I was aware of different emotions that arose in me” were rated on a 5-point Likert scale (from 1 = *Not at all* to 5 = *Very well*). The total score for the SMS has demonstrated excellent reliability (*α* = 0.95) in sample of community adults (Tanay & Bernstein, 2013). In the current paper, the internal consistency values were *α* = 0.92 for T2 and *α* = 0.91 for T4.

#### Data Analyses

Missing data, normality, and extreme outliers were evaluated (Supplement D in the Supplementary Material). Non-parametric methods were used due to failure to meet the assumption of normality and extreme outliers. Descriptive data were presented using means (standard deviations) and frequencies (percentages) of baseline characteristics at pre-intervention by experimental group (Table 1) and previous mindfulness experience (Supplement C in the Supplementary Material). The Kruskal-Wallis non-parametric test or the Fisher exact test, according to the distribution of each variable, was used to examine possible between-group differences on baseline characteristics.

The non-parametric test for examining within-group pre-post changes (Wilcoxon signed-rank) was used within the whole sample. Descriptive data were presented using means (standard deviations). Effect sizes and 95% confidence intervals of the effect sizes were reported using Cohen’s *d* statistic (small (*d* = 0.20), medium (*d* = 0.50), and large effects (*d* = 0.80)) along with the common language effect size (McGraw & Wong, 1992) as an adjusted calculation to account for the data failing to meet the assumption of normality. The common language effect size, or the probability of superiority (Grissom & Kim, 2011), expresses the likelihood that one randomly selected participant from one group would have a higher score than another randomly selected participant from a different group (Lakens, 2013). The non-parametric ANCOVA Quade’s test (Quade, 1967) was used to examine between-group differences at post-intervention across the four experimental groups, whilst controlling for the baseline scores. Estimates of the effect size (*n*_*p*_^2^) (0.01 = small, 0.06 = medium, 0.14 = large), calculated from the ANCOVA Quade’s test, were reported along with 90% confidence intervals of the effect sizes following recommendations outlined by Kline (2004). Descriptive data were also presented within each experimental group using means (standard deviations). Sensitivity analyses were conducted for between-group effects controlling for previous level of mindfulness experience (MBCT graduate versus MBP naïve), the study format (online versus in-person), and perceived impact of the Covid-19 pandemic. No differences in trait mindfulness at baseline were found across the MBCT graduate and MBP naïve groups (Supplement C in the Supplementary Material) and the four experimental groups (Table 1). Additionally, no differences in terms of perceived impact of the Covid-19 pandemic and study format (online versus face-to-face) were found across the four experimental groups. However, these variables were controlled for as covariates in sensitivity analyses to provide narrower confidence intervals or estimates of effect sizes (Supplement E2 and F2 in the Supplementary Material). To balance the issue of minimizing both Type I and II errors, adjusted effect size calculations and confidence intervals of effect sizes were prioritized over *p*-value reporting in light of the exploratory nature of the current paper (Feise, 2002).

Due to the small sample size, formal mediation methods were not used. Instead, hypotheses regarding potential mechanisms were generated by addressing key criteria (e.g., “strong association”) set forth by Kazdin (2007) in establishing a mechanism of change. This criterion was partially addressed by exploring the relationship between change in the candidate mechanisms and change in outcomes. Partial rank correlations were calculated within the whole sample and each experimental group to examine the relationship between pre-post changes in proposed mechanisms and pre-post changes in outcomes, whilst controlling for baseline levels in outcome (Conover, 1999).

### Results

#### Within-Group Effects

Within the whole sample, state-level effects in self-compassion (*d* = 0.43 (0.19, 0.68)), and pleasantness of thoughts (*d* = 0.39 (0.14, 0.63)), emotions (*d* = 0.42 (0.17, 0.66)), and body sensations (*d* = 0.86 (0.60, 1.11)) were demonstrated pre-post the first mindfulness session with small to large effect sizes (T1–T2) and pre-post the second mindfulness session but with small to medium effect sizes (*d =* 0.27 to 0.47) (T3–T4). State-level effects were also observed for mindfulness (*d* = 0.34 (0.09, 0.60)) pre-post 2 weeks of daily mindfulness training (T2–T4) with small to medium effect sizes within the whole sample. However, no state-level effects were observed for decentering (*d* = 0.24 (−0.01, 0.50)) with the confidence intervals including zero (Supplement Material E1 in the Supplementary Material).

#### Between-Group Effects

After 2 weeks of daily mindfulness practice and the second mindfulness session, whilst controlling for baseline levels, pleasantness of body sensations differed across the four experimental groups with medium to large effects (*n*_*p*_^2^ = 0.09 (0.01, 0.16), *p* = 0.017) (T3–T4). After controlling for additional covariates (i.e. previous level of mindfulness experience (MBCT graduate versus MBP naïve), perceived impact of the Covid-19 pandemic, and the format (online versus in-person)), these differences were no longer significant but were close to reaching statistical significance (*n*_*p*_^2^ = 0.08 (0.00, 0.15), *p* = 0.050). No between-group differences were detected for self-compassion, mindfulness, decentering, and pleasantness of thoughts and emotions after controlling for baseline levels and additional covariates. See Supplement Material E2 in the Supplementary Material to review all between-group effects, including descriptive data on pre-post means (standard deviations) within each experimental group.

#### The Strong Association Criterion

Within the whole sample, there were positive moderate associations between change in state self-compassion and change in pleasantness of body sensations for the first and second mindfulness sessions whilst controlling for baseline levels (T1–T2: *r*_(127)_ = 0.22 (0.05, 0.38), T3–T4: *r*_(113)_ = 0.28 (0.10, 0.44)). For the second mindfulness session, there were positive small to moderate associations between change in state self-compassion and change in pleasantness of thoughts and emotions whilst controlling for baseline levels (T3–T4: *r*_(115)_ = 0.19 (0.01, 0.36), T3–T4: *r*_(113)_ = 0.29 (0.11, 0.45)). There were also positive moderate associations between changes in state mindfulness and changes in pleasantness of body sensations and thoughts, whilst controlling for baseline levels, for the second mindfulness session (T2–T4: *r*_(113)_ = 0.22 (0.04, 0.39), T2–T4: *r*_(114)_ = 0.21 (0.03, 0.38)). These associations differed within each experimental group (see Supplement Material E3 in the Supplementary Material to review all results).

### Discussion

Study 1 has demonstrated state-level effects in candidate outcomes (e.g., pleasantness of thoughts, emotions, and body sensations) and mechanisms (i.e. self-compassion and mindfulness), within the whole sample and across multiple time points, with smaller effect sizes generally observed over time. No between-group effects were found after controlling for baseline levels and additional covariates. These preliminary results generated the hypothesis that, in the context of this sample and the explored variables, there may be more overlapping effects rather than unique effects across these four MBCT practices in terms of state-level mechanisms and outcomes. Within the whole sample, small to moderate associations between changes in state self-compassion and all outcomes were demonstrated after the second mindfulness session and moderate associations were also demonstrated between changes in state mindfulness and changes in pleasantness of body sensations and thoughts after the second mindfulness session. Another generated hypothesis was that self-compassion and mindfulness may serve as state-level mechanisms regardless of practice-type in relation to pleasantness of one’s internal experience (e.g., body sensations, thoughts, and emotions). However, in the absence of an active control group, future work will need to determine the extent to which these effects are mindfulness-specific. The key hypotheses are summarized in Supplement Material F in the Supplementary Material.

## Study 2

### Method

#### Participants

Study 2 used the same participant sample from Study 1. However, out of the original sample included at baseline (*n* = 131) for Study 1, 121 adults completed measures pre-post 2 weeks of daily training for Study 2. Out of the original sample, 10 participants (7.63%) did not provide data at post-intervention due to time conflicts (*n* = 4), no response (*n* = 4), health issues (*n* = 1), and timeline issue (*n* = 1) (Fig. 1). Over the course of 2 weeks, participants completed on average 12.07 days of daily practice out of 14 days in total (maximum 10–12 min/day or 145 min in total) (Body scan: *M* = 12.35, *SD* = 2.58; Mindful movement: *M* = 10.97, *SD* = 4.73; Breath and body: *M* = 12.35, *SD* = 3.44; Befriending: *M* = 12.62, *SD* = 2.80). No significant between-group differences were found across the four MBCT practices in terms of practice days completed (*p* = 0.550).

#### Procedure

Study 2 followed the same procedure as Study 1. However, only data points collected pre-post 2 weeks of daily practice (T1–T4) were considered for analysis. During the 2 weeks, participants were asked to use journal logs to track the amount of daily mindfulness practice (Fig. 2).

#### Measures

All measures for Study 2 were administered pre-post 2 weeks of daily mindfulness practice (T1–T4), except for the anxiety and depression measures which were administered at screening (T0) and post-intervention (T4) (Fig. 2). Psychological quality of life, depression, and anxiety were considered as trait-level outcomes. Quality of life is a broad construct that refers to the individuals’ “physical health, psychological state, level of independence, social relationship, and environment” (World Health Organization, 1996). The 6-item psychological domain of the World Health Organization Quality of Life-BREF questionnaire (WHOQOL-BREF; World Health Organization, 1996) was used to measure psychological quality of life, which evaluates positive feelings; thinking, learning, memory, and concentration; self-esteem; bodily image and appearance; and negative feelings. Items (e.g., “How much do you enjoy life?”) were answered on a 5-point Likert scale (from 1 = *Not at all* to 5 = *An extreme amount*) and total scores were transformed to the 0–100 scale (http://depts.washington.edu/seaqol/docs/Wq_bref.txt) and negative items were reverse coded. The WHOQOL-BREF (psychological domain) has demonstrated acceptable reliability (*α* = 0.75 to 0.77) across a range of studies (World Health Organization, 1996). In the current paper, the internal consistency values indicated good reliability: *α* = 0.84 for T1 and *α* = 0.85 for T4.

The 9-item Patient Health Questionnaire (PHQ-9; Kroenke et al., 2001) was used to measure depression symptoms. Items (e.g., “Over the last two weeks, how often have you been bothered by little interest or pleasure in doing things?”) were answered on a 4-point Likert scale (from 0 = *Not at all* to 3 = *Nearly every day*) and total scores were calculated. The categorical thresholds were as follows: 0–4 (none), 5–9 (mild), 10–14 (moderate), 15–19 (moderately severe), and 20–27 (severe). The PHQ-9 has demonstrated good reliability (*α* = 0.87) in a general population sample (Kocalevent et al., 2013). In the current paper, the internal consistency values indicated acceptable reliability: *α* = 0.71 for T0 and *α* = 0.62 for T4.

The 7-item Generalized Anxiety Disorder (GAD-7; Spitzer et al., 2006) questionnaire was used to measure anxiety symptoms. Items (e.g., “Over the last two weeks, how often have you been bothered by feeling nervous, anxious or on edge?”) were responded to on a 4-point Likert scale (from 0 = *Not at all* to 3 = *Nearly every day*) and total scores were calculated. The categorical thresholds were as follows: 0–4 (none), 5–9 (mild), 10–14 (moderate), and 15–21 (severe). The GAD-7 has demonstrated good reliability (*α* = 0.89) in a general population sample (Löwe et al., 2008). In the current paper, the internal consistency values were good: *α* = 0.81 for T0 and *α* = 0.86 for T4.

Self-compassion, mindfulness, decentering, attentional control, and interoceptive awareness were considered candidate trait-level mechanisms. In the current sample, changes in trait mindfulness were associated with changes in other candidate trait-level mechanisms (*r* = 0.22 to 0.43, *p* = 0.000 to 0.014) except for the noticing and emotional awareness facets of interoceptive awareness (*r* = 0.16 to 0.17, *p* = 0.062 to 0.081). The 12-item “Self-Compassion Scale Short Form” (SCS-SF; Raes et al., 2011) was used to measure trait self-compassion. Items (e.g., “I try to see my failings as part of the human condition”) were answered using a 5-point Likert scale (from 1 = *Almost never* to 5 = *Almost always*) and total mean scores were calculated and negative items were reverse coded (Neff et al., 2019). The SCS-SF has demonstrated good reliability (*α* = 0.87; Raes et al., 2011). In the current paper, the internal consistency values were also good: *α* = 0.86 for T1 and *α* = 0.86 for T4.

The 15-item “Five Facet Mindfulness Questionnaire” (FFMQ-15; Baer et al., 2006; Bohlmeijer et al., 2011) was used to measure trait mindfulness. This measure defines mindfulness as a multidimensional construct that includes several facets: Observing (noticing or attending to internal and external stimuli); Describing (labelling internal experiences); Acting with awareness (attending to the present moment); Non-judgement (taking a non-evaluative stance towards one’s internal experiences); and Non-reactivity (allowing thoughts and feelings to come and go without getting caught up in them) (Bohlmeijer et al., 2011). Items were answered using a 5-point Likert scale (from 1 = *Never or very rarely true* to 5 = *Very often or always true*) and the total and subscale scores (Observing, Describing, Acting with awareness, Non-judgement, and Non-reactivity) were calculated and negative items were reverse coded. Item examples for each subscale include Observing (e.g., “When I take a shower or a bath, I stay alert to the sensations of water on my body”); Describing (e.g., “I’m good at finding words to describe my feelings”); Acting with awareness (e.g., “I don’t pay attention to what I’m doing because I’m daydreaming, worrying, or otherwise distracted”); Non-judgement (e.g., “I believe some of my thoughts are abnormal or bad and I shouldn’t think that way”); and Non-reactivity (e.g., “When I have distressing thoughts or images I am able just to notice them without reacting”). The Observing subscale was removed from the total score based on previous findings (Gu et al., 2016) that have suggested the factor structure is unstable before and after a mindfulness intervention. Internal consistency values for the FFMQ-15 subscales have demonstrated acceptable reliability before an MBCT programme (*α* = 0.64 to 0.80) and after an MBCT programme (*α* = 0.69 to 0.83) (Gu et al., 2016). In the current paper, the internal consistency values for the total score were T1: *α* = 0.80 and T4: *α* = 0.81. The internal consistency values for subscale scores include Observing (T1: *α* = 0.68, T4: *α* = 0.68); Describing (T1: *α* = 0.80, T4: *α* = 0.81); Acting with awareness (T1: *α* = 0.80, T4: *α* = 0.79); Non-judgement (T1: *α* = 0.81, T4: *α* = 0.83); and Non-reactivity (T1: *α* = 0.75, T4: *α* = 0.76).

The 11-item “Experiences Questionnaire” (EQ; Fresco et al., 2007) was used to measure trait decentering, which has been defined as the ability to observe one’s thoughts and feelings as temporary instead of as true reflections of the self (Fresco et al., 2007). Items (e.g., “I am better able to accept myself as I am”) were answered using a 5-point Likert scale (from 1 = *Never* to 5 = *All the time*) and the total mean scores were calculated. The EQ has demonstrated good internal consistency in both undergraduate and clinical samples (*α* = 0.83 to 0.90; Fresco et al., 2007). In the current paper, the internal consistency values were *α* = 0.86 for T1 and *α* = 0.87 for T4.

The 12-item “Attentional Control Scale-Straightforward” (ACS-S; Judah et al., 2020) was used to measure trait attentional control, which broadly involves the ability to regulate attention (Derryberry & Reed, 2002). Items (e.g., “It’s very easy for me to concentrate on a difficult task when there are noises around”) were answered using a 4-point Likert scale (from 1 = *Almost never* to 4 = *Always*) and total scores were calculated. The ACS-S has demonstrated excellent internal consistency (*α* = 0.92) (Judah et al., 2020). In the current paper, the internal consistency values were T1: *α* = 0.83 and T4: *α* = 0.88.

Interoceptive awareness was measured using five subscales from the “Multidimensional Assessment of Interoceptive Awareness Version 2” questionnaire (MAIA-2; Mehling et al., 2018), based on the results of a recent study (van der Velden et al., 2023) which found pre-post changes in these subscales in an RCT comparing MBCT to a treatment-as-usual (TAU) control group. Interoceptive awareness involves the “conscious level of interoception whereby the nervous system interprets and integrates bodily signals” (Mehling et al., 2018). Items were responded to using a 6-point Likert scale (from 0 = *Never* to 5 = *Always*) and total mean scores were calculated. Item examples for each subscale include Noticing (e.g., “When I am tense I notice where the tension is located in my body”); Attention regulation (e.g., “I can pay attention to my breath without being distracted by things happening around me”); Trusting (e.g., “I am at home in my body”); Body listening (e.g., “I listen for information from my body about my emotional state”); and Emotional awareness (e.g., “I notice how my body changes when I am angry”). These five subscales of the MAIA-2 have demonstrated acceptable to good reliability (*α* = 0.64 to 0.83) with the lowest score for noticing and the highest score for the attention regulation and trusting subscales (Mehling et al., 2018). In the current paper, the internal consistency values were Noticing (*α* = 0.82 for T1, *α* = 0.72 for T4); Attention regulation (*α* = 0.91 for T1, *α* = 0.92 for T4); Trusting (*α* = 0.85 for T1, *α* = 0.87 for T4); Body listening (*α* = 0.81 for T1, *α* = 0.90 for T4); and Emotional awareness (*α* = 0.86 for T1, *α* = 0.88 for T4).

Amount of practice was calculated by the total number of journal log entries completed daily over the course of 2 weeks. For these journal logs, participants were asked to reflect on their experience whilst practicing as a manipulation check. For days where they provided written reflections, this was indicated as a day completed and days completed were tallied across the 2-week study period.

#### Data Analyses

The same data-analytic approach used in Study 1 was used for Study 2 with examining within-group and between-group effects and the strong association criterion outlined by Kazdin (2007). Additionally, Study 2 explored the gradient criterion, outlined by Kazdin (2007), which was addressed by exploring a dose-relationship in terms of the amount of self-report mindfulness practice and its association with change in the candidate mechanisms and outcomes. Partial rank correlations were calculated within the whole sample and each experimental group to examine the relationship between the amount of self-reported mindfulness practice completed and post-intervention scores in proposed mechanisms and outcomes whilst controlling for baseline levels (Conover, 1999).

### Results

#### Within-Group Effects

Within the whole sample, trait-level effects in psychological quality of life were demonstrated with small to medium effect sizes (*d* = 0.26 (0.01, 0.52), T1–T4). Trait-level effects were also observed for anxiety, with a slight increase in anxiety symptoms pre-post intervention (*d* = 0.32 (0.07, 0.57), T1–T4). However, the categorical threshold for symptom severity was indicated as no symptoms (scores of 0–4) pre-post intervention. No trait-level effects were demonstrated for depression (*d* = 0.11 (−0.14, 0.37), T1–T4). Trait-level effects were found for mindfulness (including non-reactivity and observing), decentering, self-compassion, interoceptive awareness (noticing, attentional regulation, trusting, body listening, and emotional awareness), and attentional control within the whole sample with small to large effect sizes (*d* = 0.29 to 0.64, T1–T4). Trait-level effects were not demonstrated for describing, acting with awareness, and non-judgement based on the confidence intervals including zero (Supplement G1 in the Supplementary Material).

#### Between-Group Effects

After controlling for baseline levels, no between-group differences were detected for psychological quality of life, anxiety, depression, self-compassion, decentering, attentional control, mindfulness (observing, describing, acting with awareness, non-judgement, and non-reactivity), and interoceptive awareness (noticing, attentional regulation, trusting, body listening, and emotional awareness) (*p* = 0.063 to 0.901, T1–T4). After controlling for previous levels of mindfulness experience (MBCT graduate versus MBP naïve), perceived impact of the Covid-19 pandemic, and the format (online versus in-person), these between-group differences remained non-significant. See Supplement G2 in the Supplementary Material to review all results, including descriptive data on pre-post means (standard deviations) within each experimental group.

#### The Strong Association Criterion

Within the whole sample, there were positive moderate to large associations between changes in trait self-compassion (*r*_(118)_ = 0.40 (0.24, 0.54)), mindfulness (*r*_(118)_ = 0.38 (0.22, 0.52)), acting with awareness (*r*_(118)_ = 0.22 (0.04, 0.38)), non-judgement (*r*_(118)_ = 0.35 (0.18, 0.50)), decentering (*r*_(118)_ = 0.29 (0.20, 0.45)), and body listening (*r*_(118)_ = 0.23 (0.05, 0.39)) and changes in psychological quality whilst controlling for baseline levels (T1–T4). A negative moderate association was also demonstrated within the whole sample, between change in acting with awareness and change in depression whilst controlling for baseline levels (*r*_(118)_ = −0.30 (−0.46, −0.13), T1–T4). However, the internal consistency values for the depression measure in the current study were poor to fair (T0: *α* = 0.71, T4: *α* = 0.62), and therefore, this result needs to be interpreted with caution. These associations differed within each experimental group (Supplement G3 in the Supplementary Material).

#### The Gradient Criterion

Within the whole sample, there were positive small to moderate associations between the amount of self-reported mindfulness practice and changes in noticing (*r*_(118)_ = 0.22 (0.04, 0.38)), attentional regulation (*r*_(118)_ = 0.28 (0.11, 0.44)), trusting (*r*_(118)_ = 0.22 (0.04, 0.38)), body listening (*r*_(118)_ = 0.22 (0.04, 0.38)), emotional awareness (*r*_(118)_ = 0.22 (0.04, 0.38)), mindfulness (*r*_(118)_ = 0.18 (0.00, 0.35)), self-compassion (*r*_(118)_ = 0.18 (0.00, 0.35)), decentering (*r*_(118)_ = 0.23 (0.05, 0.39)), attentional control (*r*_(118)_ = 0.26 (0.08, 0.42)), and psychological quality of life (*r*_(118)_ = 0.25 (0.07, 0.41)), whilst controlling for baseline levels (T1–T4). These associations differed within each experimental group (Supplement Material G4 in the Supplementary Material).

### Discussion

Study 2 demonstrated trait-level effects in proposed outcomes (i.e., psychological quality of life and anxiety), within the whole sample. The slight increase in anxiety may be due to a floor effect given that the sample reported no symptoms of anxiety pre-post intervention (scores 0–4). Trait-level effects were also demonstrated in candidate mechanisms (i.e., self-compassion, decentering, mindfulness, attentional control, and interoceptive awareness), except for describing, acting with awareness, and non-judgement. No between-group effects were demonstrated pre-post 2 weeks of mindfulness training. These preliminary findings build on the findings of Study 1 and suggest that, in the context of this sample and the variables explored, there may be more overlapping effects rather than unique effects across these four MBCT practices in terms of trait-level mechanisms and outcomes. Within the whole sample, changes from pre- to post-intervention in mindfulness, self-compassion, decentering, and interoceptive awareness (i.e., body listening) were associated with psychological quality of life post-intervention, after controlling for baseline levels, and the amount of self-reported practice was also associated with these variables post-intervention after controlling for baseline levels. In light of these findings, an additional hypothesis generated was that mindfulness, self-compassion, decentering, and interoceptive awareness (i.e. body listening) may serve as trait-level mechanisms regardless of practice type. However, as pointed out in Study 1, future work will need to test these hypotheses in the context of an RCT with an active control arm to disentangle the extent to which these effects are mindfulness-specific. The key hypotheses are summarized in Supplement H in the Supplementary Material.

## General Discussion

The aim of the current paper was to generate hypotheses regarding the state-level and trait-level effects and potential mechanisms of four MBCT practices (body scan, mindful movement, breath and body, and befriending). The results of Study 1 and 2 generated the hypothesis that these four MBCT mindfulness practices may produce both state-level and trait-level effects outside the framework of the M-FP programme and that these four practices may share more overlapping than unique effects. Study 1 found state-level effects in outcomes (pleasantness of thoughts, emotions, and body sensations) and candidate mechanisms (state self-compassion and mindfulness) after the first and second mindfulness sessions, with smaller effect sizes demonstrated over time. Study 2 found trait-level effects in psychological quality of life and candidate mechanisms (trait self-compassion, decentering, mindfulness, attentional control, and interoceptive awareness) after 2 weeks of daily mindfulness practice. Overall, no between-group effects were observed, which suggests that the choice of practice may not matter when it comes to targeting key mechanisms of change and outcomes in the context of the proposed study design and sample. This finding is supported by a recent study (Fincham et al., 2023), with a similar experimental design, that compared a movement meditation to a sitting practice (with different doses) and reported no between-group differences in terms of changes in well-being, distress, and mindfulness. Future research may want to consider other formats; for instance, more intensive interventions to examine whether effects become more specific over time with more practice and future work will need to determine the extent to which these effects are mindfulness-specific. Other studies (Sauer-Zavala et al., 2013; Singer & Kok, 2017) have demonstrated that different mindfulness practices may simultaneously produce overlapping and unique effects. Therefore, the lack of specific effects reported may be due to lack of statistical power and future research may wish to further investigate these effects in a larger sample. Conversely, future research may wish to investigate other components of MBCT (e.g., teacher inquiry, group-effect) to understand additive benefits.

Another hypothesis generated was that self-compassion, mindfulness, decentering, and interoceptive awareness (i.e. body listening) may serve as key mechanisms of change for these four MBCT practices. In the current paper, two key criteria in establishing a mechanism (“strong association” and “gradient”) were addressed using Kazdin’s (2007) framework. After the second mindfulness session, Study 1 demonstrated small to moderate associations between change in state self-compassion and mindfulness and change in outcome (e.g., pleasantness of thoughts and body sensations). Study 2 demonstrated small to moderate associations between the amount of self-reported mindfulness practice and candidate mechanisms (self-compassion, mindfulness, decentering, and interoceptive awareness (i.e. body listening)) and small to large associations between change in these candidate mechanisms and outcome (psychological quality of life) post-intervention, after controlling for baseline levels. These variables (i.e. self-compassion, mindfulness, decentering) have been indicated as key mechanisms in the context of the entire MBCT programme (Alsubaie et al., 2017; Feldman & Kuyken, 2019; Gu et al., 2015; van der Velden et al., 2015). In light of this, future work may wish to explore how to sustain this learning beyond the traditional 8-week MBCT programmes, which is an area of research that is currently underdeveloped.

One unexpected finding was that these mindfulness practices, outside the framework of M-FP and in the context of a low-dose 2-week intervention, did not promote improvements in mental health symptoms. The current paper reported no changes in depression and a small increase in anxiety symptoms pre-post 2 weeks of daily practice within the whole sample. Given that the sample was well in terms of categorical thresholds, a slight increase in anxiety symptoms may be due to a possible floor effect, in that there was not much room for improvement. This effect may also be explained by a potential moderating effect of baseline anxiety levels or regression to the mean effect. Exploratory sub-group analyses of extreme outliers (Supplement Material D in the Supplementary Material) support this assumption with the finding that those with higher baseline levels of anxiety and depression generally improved whereas those with lower baseline levels generally deteriorated post-intervention. However, another possible reason may be that the intervention was not long enough to produce positive effects in mental health. A recent systematic review (Blanck et al., 2018) demonstrated small to medium effects of stand-alone mindfulness practices (e.g., body scan, sitting meditation) on mental health outcomes (i.e. depression and anxiety) with an average treatment dosage of 372 min, whereas our maximum treatment dosage was 145 min. Anecdotally, we see in traditional 8-week MBPs that some people initially get worse as they become more aware of their thoughts and emotions and then around Week 4 start to improve. Future research may wish to explore outcomes in more intensive interventions (e.g., 4- or 8-week programmes) in a sample with a wider range of mental health symptoms to disentangle these effects. Ultimately, in the context of null hypothesis testing, we cannot determine whether the absence of evidence for finding improvements in mental health is evidence for absence and this should therefore be clarified in future research.

Past research has provided outcome-only (Britton et al., 2018; Sauer-Zavala et al., 2013) and mediation studies of unique mindfulness practices or techniques (e.g., Dambrun et al., 2019; Hayes-Skelton & Lee, 2020; McClintock & Anderson, 2015; Villa & Hilt, 2014). However, these studies differ in terms of the sample or condition explored, the intention behind the MBP (e.g., treatment, prevention, promotion), and dosage. Studies (Dambrun et al., 2019; Villa & Hilt, 2014) that are most similar to the current paper, in terms of intention (e.g., promotion) and sample, differ in terms of the constructs explored and dosage, which makes it difficult to consolidate findings. Ultimately, future work needs to focus on replication and more work is required to reach a consensus on the operational definitions of the candidate mechanisms and outcomes in relation to the proposed theory of change. Ultimately, future research can use the logic diagrams generated (Supplement F and H in the Supplementary Material) as a guide for hypothesis testing.

### Limitations and Future Research

Some strengths of the current paper include the theory and evidence-driven candidate mechanisms and outcomes explored and the covariate-adjusted sensitivity analyses to control for variables (e.g., previous mindfulness experience, study format, and perceived impact of the Covid-19 pandemic) that could have influenced the results. Additional strengths include the use of randomization and the relatively inclusive eligibility criteria to recruit individuals from the community, interested in mindfulness, with a range of mindfulness experience. In terms of limitations, the small sample size and subsequent lack of power limited our ability to detect a true effect, especially if the true effect was small. Moreover, multiple comparisons could have increased the probability of chance findings. Given that this research is still in its early stages, the authors considered an exploratory design to generate hypotheses for future research to test. Although not adjusted for multiple comparisons, confidence intervals of the effects with adjusted effect size calculations (e.g., common language effect size) were prioritized to help balance Type I and Type II errors. In light of the exploratory nature of the current study, and limited pre-existing data on the effects and potential mechanisms of these four MBCT practices, an a priori power calculation was not conducted. Moreover, in light of post hoc power calculations being informed by *p*-values and therefore limited in terms of interpretability in the context of an exploratory study, the current study emphasized effect sizes for future work to use to inform an a priori power calculation (Hoenig & Heisey, 2001). Other limitations include the self-selective sample, which limits generalizability, and the lack of an active control group, which limits interpretation of MBCT-specific effects. Past research (Goldberg, 2022) has recognized the difficulty in identifying an appropriate control group and dosage for mindfulness interventions which provides support for conducting more dismantling trials, like the current paper, to disentangle effects of key MBCT components to isolate active ingredients. Moreover, although efforts were made to minimize potential bias (e.g., a researcher outside the core team created the randomization list), having the same researcher involved in the data collection and analyses was a limitation. Lastly, the administration of the state decentering and mindfulness measures after each single mindfulness session (T2, T4) could be measuring trait-level changes. Changes in state-level variables were significantly associated with changes in trait-level variables for both mindfulness and decentering with moderate to large correlations (*r* = 0.39 to 0.43; Supplement I in the Supplementary Material). Based on these chosen time points for administration, it is difficult to discern the extent to which these variables are measuring state- versus trait-level changes. Lastly, although the VAS used for evaluating pleasantness of thoughts, emotions, and body sensations were theoretically driven and adapted from past research, the lack of available psychometric information was a limitation.

Future work may wish to explore the overlapping and unique effects of MBCT practices in the context of more intensive interventions, with additional time points, and in a larger sample with a wider distribution of mental health symptoms. Additionally, future work may wish to consider adapting the state-level measures for decentering and mindfulness to be used pre-post single mindfulness sessions instead of post-only. In terms of investigating formal mediation, the hope is that the generated hypotheses from the current paper can inform future work. The authors of the current paper recommend future studies to calculate indirect effects, with a larger sample size, following a framework outlined by Zhao et al. (2010). This framework addresses limitations of the commonly used Baron and Kenny method and can supplement some of the Kazdin criteria (Zhao et al., 2010). At the moment, MBPs are being adapted rapidly in different populations and contexts but the evidence base is lagging far behind (Creswell, 2017). Exploratory work can help drive more rigorous investigations so that adaptations are driven by the evidence and consequently more appropriately tailored (Loucks et al., 2022). We know that interventions that are offered to the general population tend to be more accessible but produce smaller effect sizes (Greenberg & Abenavoli, 2017), so an investigation of its constituent components in terms of how and why they work can further our understanding to best optimize both accessibility and effects.

### Supplementary Information

ESM 1 (DOCX 410 kb)

## Data Availability

The data generated from this study will be made available upon reasonable request to researchers who provide a methodologically sound proposal and whose proposed use of the data is aligned with the aims of the original study and where the proposed use has been approved by an independent ethical review committee. Please contact the corresponding author, SM, for such requests. Due to ethical constraints, the amount of data that can be shared may depend on the request. All relevant results are provided as supplementary material and the relevant stimuli and materials are publicly available and cited within the text. This study was not formally pre-registered but the research questions, methods, and analytical strategies were outlined a priori in an ethics application which can also be shared upon request.
