# Supplementary material for: The State- and Trait-Level Effects and Candidate Mechanisms of Four Mindfulness-Based Cognitive Therapy (MBCT) Practices: Two Exploratory Studies
Source: Mindfulness (N Y). 2023 Aug 24;14(9):2155–71. doi: 10.1007/s12671-023-02193-6 (PMC10545571; doi:10.1007/s12671-023-02193-6)
Supplement: Supplementary file 1 — (DOCX 410 kb) [file 12671_2023_2193_MOESM1_ESM.docx]

**The state- and trait-level effects and candidate mechanisms of four Mindfulness-Based Cognitive Therapy (MBCT) practices: Two exploratory studies**

*Supplementary Information*

**Contents**

**Supplement A**: Description of MBCT practices…………………………….…………..……………….…1

**Supplement B**: Overall Logic Diagram for Study 1 and 2……………………………………..…...………2

**Supplement C:** Descriptive statistics of whole sample and group by level of mindfulness experience at baseline………………………………………………………………………………………………………3

**Supplement D:** Missing data, extreme outliers, and normality…………………………………………......5

**Supplement E1:** Study 1 Results – Within-group effects..............................................................................9

**Supplement E2:** Study 1 Results – Between-group effects………………………………………..........…13

**Supplement E3**: The strong association criterion – Study 1 ………………………………………….......19

**Supplement F**: Logic Diagram for Study 1……………………………………………………………......24

**Supplement G1:** Study 2 Results – Within-group effects....................................................... …………....25

**Supplement G2:** Study 2 Results – Between-group effects.....................................................................…27

**Supplement G3**: The strong association criterion –Study 2………………………………………......…...33

**Supplement Material G4**: The gradient criterion – Study 2…………………………………………........43

**Supplement H**: Logic Diagram for Study 2…………………………………………………………..…...48

**Supplement I:** Additional information – The association between pre-post scores for state and trait decentering and mindfulness……………………………………………………………………………….49

**Supplement J:** References for Supplementary Material…………………………………………..………50

1. **Description of MBCT practices**

| **Week** | **Formal practice** | **Description** | **Instruction** |
| --- | --- | --- | --- |
| 1 | Breath and body* | Recognising autopilot; sensing the internal weather patterns with the breath; stabilising attention | Twice daily for six days |
| 2 | Body scan* | Reintegrating mind and body; seeing the mind’s reactivity by learning to pay attention to the body | Twice a day for six days |
| 3 | Mindful movement* | Anchoring awareness in the moving body; becoming aware of striving and recognizing your limits | Mindful movement followed by breath and body practice daily for six days |
| 4 | Sounds and thoughts | Receiving thoughts, along with their mental associations, as they come; recognizing thoughts for what they are | Breath and body followed by sounds and thoughts twice daily for six days |
| 5 | Exploring difficulty | Learning to respond to difficulty in a skilful way; noticing the temptation to suppress negative experiences and to then turn towards them with acceptance | Breath and body and sounds and thoughts followed by exploring difficulty daily for six days |
| 6 | Befriending* | Learning how to treat yourself and others with kindness; growing to love, respect and honour yourself; letting go of the past and sense of permanency | Daily for six days with optional breath practice (e.g., body and breath) |
| 7 | Choice of practice | Understanding which activities are depleting and nourishing; cultivating a different relationship to the world for the better | Practice two practices daily for six days |
| 8 | Choice of practice | Understanding how to weave these skills into your daily life; deciding for yourself which practices you need and how long to practice | Not applicable. |

This table provides the original instructions and descriptions for each formal practice within an eight-week M-FP programme. * These practices were explored in the current study.

1. **Logic Diagram – Study 1 and 2**


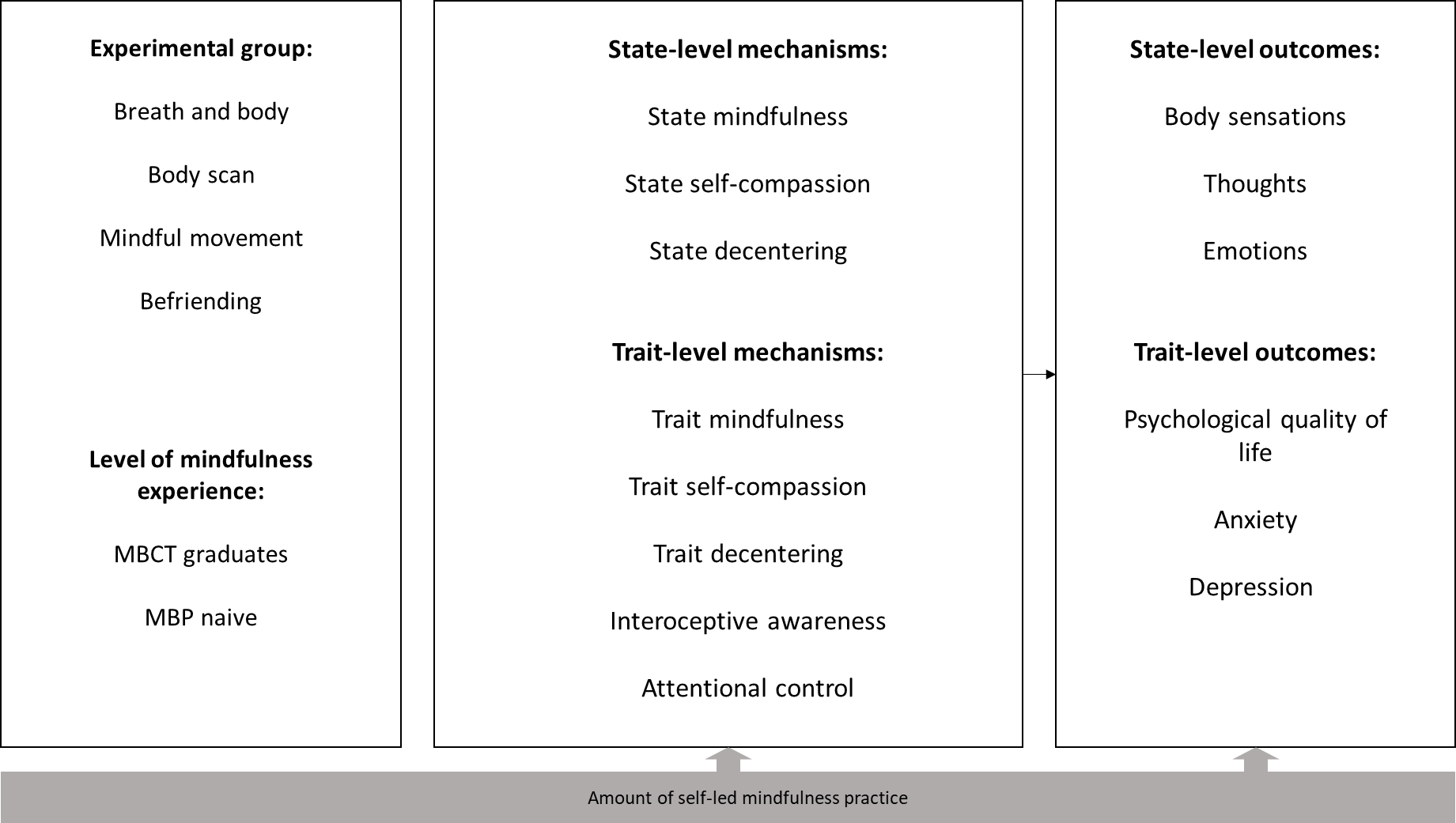


This logic diagram provides a simplified visual of the key components (MBCT practices, candidate mechanisms, and outcomes) that were explored across Study 1 and 2. The arrows indicate the relationships that were explored (e.g., the association between change in candidate mechanisms and outcomes and amount of self-led mindfulness practice and candidate mechanisms and outcomes). This logic diagram was used as a guide in the development of the study design for Study 1 and 2 and the aim of this paper is to generate specific hypotheses from this diagram that can be tested in future research with a larger sample size.

1. **Descriptive statistics of whole sample and group by level of mindfulness experience at baseline**

|  | **Whole sample**  **(*N* = 131)** | **MBCT Graduates**  **(*n* = 63)** | **MBP Naive**  **(*n* = 68)** | ***p*** |
| --- | --- | --- | --- | --- |
| **Gender, female** (frequency) | 99 | 50 | 49 | 0.477 |
| **%** | 75.60 | 79.4 | 72.1 |  |
| **Age,** *M (SD)* | 41.46 (15.20) | 46.29 (14.19) | 36.99 (14.83) | < 0.001 |
| Range | 18-75 | 21-74 | 18-75 |  |
| **Depression** *M (SD)* | 3.18 (2.98) | 2.87 (2.87) | 3.47 (3.08) | 0.292 |
| Range | 0.00-14.00 | 0.00-14.00 | 0.00-11.00 |  |
| **Anxiety** *M (SD)* | 3.69 (3.23) | 3.44 (3.07) | 3.93 (3.38) | 0.448 |
| Range | 0.00-14.00 | 0.00-14.00 | 0.00-14.00 |  |
| **Psych QOL** *M (SD)* | 65.11 (12.98) | 65.28 (13.20) | 64.95 (12.86) | 0.612 |
| Range | 25.00-100.00 | 25.00-87.50 | 25.00-100.00 |  |
| **Mindfulness** *M (SD)* | 40.27 (6.38) | 41.17 (6.57) | 39.44 (6.12) | 0.147 |
| Range | 25.00-57.00 | 25.00-57.00 | 29.00-56.00 |  |
| **Observing** *M (SD)* | 9.98 (2.44) | 10.06 (2.40) | 9.91 (2.50) | 0.518 |
| Range | 4.00-15.00 | 4.00-15.00 | 5.00-15.00 |  |
| **Describing** *M (SD)* | 10.76 (2.42) | 11.27 (2.44) | 10.29 (2.32) | 0.022 |
| Range | 5.00-15.00 | 5.00-15.00 | 6.00-15.00 |  |
| **Acting with awareness** *M (SD)* | 9.07 (2.28) | 9.03 (2.11) | 9.10 (2.45) | 0.920 |
| Range | 3.00-15.00 | 4.00-14.00 | 3.00-15.00 |  |
| **Non-judging** *M (SD)* | 11.30 (2.71) | 11.48 (2.77) | 11.13 (2.66) | 0.359 |
| Range | 4.00-15.00 | 4.00-15.00 | 4.00-15.00 |  |
| **Non-reactivity** *M (SD)* | 9.15 (2.33) | 9.40 (2.35) | 8.91 (2.30) | 0.228 |
| Range | 3.00-15.00 | 5.00-14.00 | 3.00-15.00 |  |

This table includes descriptive data of the sample by level of mindfulness experience at baseline using a complete-cases approach. Means (standard deviations) or frequencies (percentages) of baseline characteristics are reported. The one-way analysis of variance for non-parametric tests (Kruskal-Wallis) or the corresponding Fisher Freeman Halton Exact Test was used to examine possible between-group differences at baseline. Depression was measured using the PHQ-9 (scores range from 0 to 27 with symptom severity cut-offs at 5 (mild), 10 (moderate), 15 (moderately severe), and 20 (severe)) (Kroenke et al., 2001). Anxiety was measured using the GAD-7 (scores range from 0-21 with symptom severity cut-offs at 5 (mild), 10 (moderate), 15 (severe)) (Spitzer et al., 2006). Psychological quality of life was measured using the psychological domain of the WHOQOL-BREF (scores range from 0-100; scores of 60 and above are interpreted as optimal (Silva et al., 2014). Mindfulness was measured using the FFMQ-15 (scores range from 12-60 [with the observing scale omitted from total score]; the total score with the Observing subscale included was 50.26 (*SD* = 7.62) [scale range from 15-75] within the whole sample at baseline; in a selected sample of secondary school teachers the average score was 51.5 (*SD* = 6.8) using all five subscales for the total score (Montero-Marin et al., 2021); in a selected sample of mostly individuals who have completed a formal mindfulness-based programme the average score was 52.66 (*SD* = 9.14) using only four subscales [omitting the Observing subscale] (J. M. G. Williams et al., 2022). Trait-level outcomes and the primary trait-level mechanism (mindfulness along with the subscales) were prioritized to minimise chance findings due to small sample size. The observing and non-reactivity were prioritized when looking at differences across these groups based on past research which found that experienced meditators had significantly higher scores for these subscales compared to meditation naïve individuals (Thompson & Waltz, 2007). Descriptively, we can see that the MBP naïve individuals were slightly younger (*M* = 36.99, *SD* = 14.83) than the MBCT graduate individuals (*M* = 46.29, *SD* = 14.19). However, given that there were no differences in trait mindfulness across groups, and to minimise multiple testing, no sub-group analyses were conducted by level of previous mindfulness experience. In the current study, for those that identified as MBCT naïve (*n* = 68), 55.9% of them reported that they had some other mindfulness experience (e.g., digital platforms such as Headspace or Calm), which may explain why we are not seeing differences across these groups. In the current study, MBCT graduates were asked what type of MBCT programme they completed prior to the start of the course and 20.6% reported they completed MBCT for Life (MBCT-L) (Strauss et al., 2021); 12.7% reported they completed MBCT for Depression (Segal et al., 2018); 9.5% reported they completed MBCT Finding Peace in a Frantic World (M-FP) (M. Williams & Penman, 2011); 7.9% reported they completed a follow-up programme for MBCT graduates called MBCT Taking it Further (MBCT-TiF); 9.5% reported ‘other’; and 25.4% reported they were ‘unsure’.

1. **Missing data, extreme outliers, and normality**

Due to a small sample size and subsequent lack of power, a formal test for missingness (e.g., Little’s Missing Completely at Random Test; MCAR) was not conducted. However, missing data was evaluated by comparing descriptive statistics on gender, age, previous mindfulness experience, and baseline depression, anxiety, psychological quality of life, and mindfulness scores between the sample that was lost by post-intervention and the sample that remained by post-intervention. Please see the table below for the means (SD) or frequencies of baseline characteristics in those that were lost at post-intervention and remained in the study at post-intervention. Descriptively, the sample that remained by post-intervention had a lower percentage of female-identifying individuals, was slightly younger, and had a higher percentage of individuals that were naïve to taking part in a formal mindfulness-based programme. In terms of baseline age, anxiety, depression, psychological quality of life, no meaningful difference across groups seem to be detected. Overall, more female-identifying individuals dropped than male-identifying individuals by post-intervention, which may just reflect the majority of the sample overall identifying as female. The data suggests that more individuals that completed an MBCT programme [MBCT graduates] prior to the start of the study dropped out than the naïve group, so the data at post-intervention may be biased to a group comprised of more naïve individuals. However, in Supplementary C we see that there may not be meaningful differences between the naïve and graduate groups.

Exploratory sub-group analyses were performed to investigate extreme outliers on outcomes of anxiety and depression pre-post intervention (*n* = 121). Extreme outliers (calculated as 3rd quartile + 3* interquartile range and 1^st^ quartile – 3*interquartile range) that experienced improvement in their anxiety scores pre-post intervention generally reported higher baseline levels of anxiety (*M* = 10.60, *SD* = 2.97, *n* = 5) and depression (*M* = 8.40, *SD* = 3.98 *n* = 5) compared to those that experienced deterioration [Anxiety: *M* = 2.67, *SD* = 2.81, *n* = 6; Depression: *M* = 2.33, *SD* = 3.93, *n* = 6]. Extreme outliers that experienced improvement in their depression scores pre-post intervention generally reported higher baseline levels of anxiety (*M* = 6.43, *SD* = 5.06, *n* = 7) and depression (*M* = 9.86, *SD* = 2.41, *n* = 7) compared to those that experienced deterioration [Anxiety: *M* = 4.67, *SD* = 2.92, *n* = 9; Depression: *M* = 1.44, *SD* = 1.42, *n* = 9]. Due to the exploratory nature of this study, extreme observations were included in the final analysis. Non-parametric methods based on ranks were used, as they are more robust to such outliers, non-normal distributions, and smaller sample sizes. Please see below tables testing for normality of the pre-post scores and the differential scores using the Kolmogorov-Smirnov test.

**Selected participant baseline characteristics by status post-intervention**

|  | **Participants lost at post-intervention** | **Remaining participants** |
| --- | --- | --- |
|  | **Total**  **(*n* = 10)** | **Total**  **(*n* = 121)** |
| **Gender, female** (frequency) | 9 | 90 |
| **%** | 90.00 | 74.40 |
| **Age,** *M (SD)* | 47.00 (12.53) | 41.00 (15.36) |
| Range | 24-66 | 18-75 |
| **Mindfulness experience, MBCT naïve** (frequency) | 3 | 65 |
| % | 30.00 | 53.70 |
| **Depression** *M (SD)* | 3.70 (4.03) | 3.14 (2.90) |
| Range | 0.00-11.00 | 0.00-14.00 |
| **Anxiety** *M (SD)* | 3.44 (4.03) | 3.71 (3.19) |
| Range | 0.00-13.00 | 0.00-14.00 |
| **Psych QOL** *M (SD)* | 68.75 (8.39) | 64.81 (13.26) |
| Range | 54.17-83.33 | 25.00-100.00 |
| **Mindfulness** *M (SD)* | 40.20 (3.91) | 40.28 (6.55) |
| Range | 34.00-48.00 | 25-57 |

This table shows the means (*SD*) or frequencies of baseline characteristics in those that were lost at post-intervention and remained in the study at post-intervention, using a complete-case approach, to descriptively explore the extent to which missingness is at random. For anxiety, one case was missing in both the sample that was lost post-intervention and the sample that remained in the study at post-intervention. Looking at this data descriptively, it does not look like meaningful information was lost in terms of symptoms of depression and anxiety (which remained in the ‘none’ range; scores 0-4) and psychological quality of life (which remained in optimal ranges) across groups, and minimal differences for mindfulness scores. No meaningful differences seem to be detected for age although the sample that remained post-intervention was slightly younger. In terms of gender, there was a smaller proportion of females but the majority of the sample still identified as female. A larger proportion of naïve-identifying individuals were found in the sample that remained but, in other analyses, we were able to show that there were no meaningful differences across the naïve and graduate groups (see Supplement C).

**Kolmogorov-Smirnov - Normality – Pre-test and Post-test Scores**

| **Variable** | **Pre-test statistic (df)** | **Sig.** | **Post-test statistic (df)** | **Sig.** |
| --- | --- | --- | --- | --- |
| Pleasantness of body sensations (T1-T2) | 0.16 (131) | p < 0.001 | 0.13 (130) | p <0.001 |
| Pleasantness of body sensations (T3-T4) | 0.10 (116) | p =0 .01 | 0.11 (118) | p = 0.002 |
| Pleasantness of body sensations (T2-T4) | 0.10 (117) | p = 0.004 | 0.11 (118) | p =0.002 |
| Pleasantness of emotions (T1-T2) | 0.16 (128) | p < 0.001 | 0.11 (128) | p < 0.001 |
| Pleasantness of emotions (T3-T4) | 0.08 (116) | p = 0.05 | 0.11 (118) | p < 0.001 |
| Pleasantness of emotions (T2-T4) | 0.13 (115) | p = 0.000 | 0.11 (118) | p = 0.001 |
| Pleasantness of thoughts (T1-T2) | 0.20 (131) | p < 0.001 | 0.16 (131) | p < 0.001 |
| Pleasantness of thoughts (T3-T4) | 0.19 (118) | p < 0.001 | 0.15 (118) | p < 0.001 |
| Pleasantness of thoughts (T2-T4) | 0.15 (118) | p < 0.001 | 0.15 (118) | p < 0.001 |
| State self-compassion (T1-T2) | 0.08 (131) | p = 0.03 | 0.10 (131) | p = 0.010 |
| State self-compassion (T3-T4) | 0.08 (118) | p = 0.08 | 0.13 (118) | p = 0.001 |
| State mindfulness (T2-T4) | 0.10 (117) | p = 0.01 | 0.06 (118) | p = 0.200 |
| State decentering (T2-T4) | 0.07 (117) | p = 0.17 | 0.10 (118) | p = 0.010 |
| Trait self-compassion | 0.07 (121) | p = 0.20 | 0.06 (121) | p = 0.200 |
| Trait mindfulness | 0.06 (121) | p = 0.20 | 0.07 (121) | p = 0.200 |
| *Observing* | 0.11 (121) | p < 0.001 | 0.14 (121) | p < 0.001 |
| *Describing* | 0.10 (121) | p = 0.003 | 0.12 (121) | p < 0.001 |
| *Acting with awareness* | 0.14 (121) | p < 0.001 | 0.15 (121) | p <0.001 |
| *Non-judgement* | 0.13 (121) | p < 0.001 | 0.11 (121) | p < 0.001 |
| *Non-reactivity* | 0.10 (121) | p = 0.004 | 0.12 (121) | p < 0.001 |
| Noticing | 0.10 (121) | p =0.004 | 0.10 (121) | p = 0.010 |
| Attentional regulation | 0.07 (121) | p = 0.20 | 0.10 (121) | p = 0.010 |
| Trusting | 0.09 (121) | p = 0.02 | 0.14 (121) | p < 0.001 |
| Body listening | 0.11 (121) | p < 0.001 | 0.12 (121) | p < 0.001 |
| Emotional awareness | 0.08 (121) | p = 0.05 | 0.17 (121) | p < 0.001 |
| Attentional control | 0.07 (121) | p = 0.20 | 0.08 (121) | p = 0.040 |
| Trait decentering | 0.07 (121) | p = 0.20 | 0.08 (121) | p = 0.040 |
| Anxiety | 0.19 (120) | p < 0.001 | 0.17 (120) | p < 0.001 |
| Depression | 0.16 (121) | p < 0.001 | 0.13 (121) | p < 0.001 |
| Psychological quality of life | 0.11 (121) | p = 0.001 | 0.08 (121) | p = 0.040 |

This table includes the Kolmogorov-Smirnov test statistic and significance values for all pre-test and post-test variables within the whole sample using a complete-case approach.

**Kolmogorov-Smirnov - Normality – Differential Scores**

|  | **Test statistic (df)** | **Sig.** |
| --- | --- | --- |
| Pleasantness of body sensations (T1-T2) | 0.08 (130) | p = 0.053 |
| Pleasantness of body sensations (T3-T4) | 0.08 (116) | p = 0.080 |
| Pleasantness of emotions (T1-T2) | 0.14 (128) | p < 0.001 |
| Pleasantness of emotions (T3-T4) | 0.11 (116) | p = 0.001 |
| Pleasantness of thoughts (T1-T2) | 0.11 (131) | p = 0.001 |
| Pleasantness of thoughts (T3-T4) | 0.12 (118) | p < 0.001 |
| State self-compassion (T1-T2) | 0.10 (131) | p = 0.003 |
| State mindfulness (T2-T4) | 0.06 (117) | p = 0.200 |
| State decentering (T2-T4) | 0.08 (117) | p = 0.056 |
| Pleasantness of body sensations (T2-T4) | 0.08 (117) | p = 0.100 |
| Pleasantness of emotions (T2-T4) | 0.11 (115) | p = 0.002 |
| Pleasantness of thoughts (T2-T4) | 0.13 (118) | p < 0.001 |
| Trait self-compassion | 0.09 (121) | p = 0.016 |
| Trait mindfulness | 0.11 (121) | p = 0.001 |
| *Observing* | 0.12 (121) | p < 0.001 |
| *Describing* | 0.15 (121) | p < 0.001 |
| *Acting with awareness* | 0.16 (121) | p < 0.001 |
| *Non-judgement* | 0.14 (121) | p < 0.001 |
| *Non-reactivity* | 0.12 (121) | p < 0.001 |
| Noticing | 0.11 (121) | p = 0.002 |
| Attentional regulation | 0.07 (121) | p = 0.200 |
| Trusting | 0.11 (121) | p = 0.001 |
| Body listening | 0.12 (121) | p < 0.001 |
| Emotional awareness | 0.11 (121) | p = 0.002 |
| Attentional control | 0.08 (121) | p = 0.060 |
| Trait decentering | 0.10 (121) | p = 0.008 |
| Anxiety | 0.15 (120) | p < 0.001 |
| Depression | 0.12 (121) | p < 0.001 |
| Psychological quality of life | 0.12 (121) | p < 0.001 |

This table includes the Kolmogorov-Smirnov test statistic and significance values for all difference (pre-post) in scores variables within the whole sample using a complete-case approach.

1. **Study 1 results**
2. **Within-group effects**

**Within-group analyses (whole sample) for state-level effects pre-post the first single mindfulness session (Study 1, T1-T2)**

|  | **Whole sample (*n* = 131)** | | | | | | |
| --- | --- | --- | --- | --- | --- | --- | --- |
|  | | **Pre M**  **(SD)** | **Post M**  **(SD)** | **Cohen’s *d* with CI** | **CLES** | ***n*** | ***p*** |
| **Perceived pleasantness of body sensations** | | 44.48  (18.12) | 59.52  (17.04) | 0.86 [0.60, 1.11] | 0.73% | 130* | < 0.001 |
| **Perceived pleasantness of emotions** | | 53.94  (17.78) | 62.06  (20.95) | 0.42 [0.17, 0.66] | 0.62% | 128* | < 0.001 |
| **Perceived pleasantness of thoughts** | | 54.12  (17.33) | 60.69  (16.39) | 0.39 [0.14, 0.63] | 0.61% | 131 | < 0.001 |
| **State self-compassion** | | 64.89  (19.65) | 72.87  (17.36) | 0.43 [0.19, 0.68] | 0.62% | 131 | < 0.001 |

This table depicts the within-group analyses (whole sample) for state-level effects pre-post the first mindfulness session using a complete-case approach. Perceived pleasantness of body sensations, emotions, and thoughts, and state self-compassion (SSC) scores were reported before (T1; ‘Pre’) and after (T2; ‘Post’) the first mindfulness session. The non-parametric test for examining within-group pre-post changes (Wilcoxon Signed-Rank) was used and effect sizes with confidence intervals for effect size were reported using Cohen’s *d* along with the adjusted calculation using common language effect size (CLES), using an online calculator to transform Cohen’s *d* to CLES: https://www.psychometrica.de/effect_size.html. Cohen’s *d* conventions for small (0.2), medium (0.5), and large effects (0.8) were used for interpretation.*Cases were excluded if participants indicated that they experienced no body sensations, emotions, or thoughts. For pleasantness of body sensations, there was one case missing from the post scores (*n* = 130). For pleasantness of emotions, there were three cases missing from the pre- and post-scores (*n* = 128).

**Within-group analyses (whole sample) for state-level effects pre-post the second single mindfulness session (Study 1, T3-T4)**

|  | **Whole sample (*n* = 118)** | | | | | | |
| --- | --- | --- | --- | --- | --- | --- | --- |
|  | | **Pre M**  **(SD)** | **Post M**  **(SD)** | **Cohen’s *d* with CI** | **CLES** | ***n*** | ***p*** |
| **Perceived pleasantness of body sensations** | | 51.37  (20.22) | 61.19  (21.38) | 0.47 [0.21, 0.73] | 0.63% | 116* | < 0.001 |
| **Perceived pleasantness of emotions** | | 55.12  (18.72) | 61.85  (18.03) | 0.37 [0.11, 0.63] | 0.60% | 116* | < 0.001 |
| **Perceived pleasantness of thoughts** | | 53.47  (17.30) | 58.46  (19.13) | 0.27 [0.02, 0.53] | 0.58% | 118 | < 0.001 |
| **State self-compassion** | | 71.53  (15.53) | 76.72  (15.56) | 0.33 [0.08, 0.59] | 0.59% | 118 | < 0.001 |

This table depicts the within-group analyses (whole sample) for state-level effects pre-post the second mindfulness session using a complete-case approach. Perceived pleasantness of body sensations, emotions, and thoughts, and state self-compassion (SSC) scores were reported before (T3; ‘Pre’) and after (T4; ‘Post’) the second mindfulness session. The non-parametric test for examining within-group pre-post changes (Wilcoxon Signed-Rank) was used and effect sizes with confidence intervals for effect size were reported using Cohen’s *d* along with the adjusted calculation using common language effect size (CLES), using an online calculator to transform Cohen’s *d* to CLES: https://www.psychometrica.de/effect_size.html. Cohen’s *d* conventions for small (0.2), medium (0.5), and large effects (0.8) were used for interpretation.*Cases were excluded if participants indicated that they experienced no body sensations, emotions, or thoughts. For pleasantness of body sensations, there were two cases missing from the pre-scores (*n* = 116). For pleasantness of emotions, there were two cases missing from the pre-scores (*n* = 116).

**Within-group analyses (whole sample) for state-level effects pre-post two weeks of daily mindfulness training (Study 1, T2-T4)**

|  | **Whole sample (*n* = 118)** | | | | | | |
| --- | --- | --- | --- | --- | --- | --- | --- |
|  | | **Pre M**  **(SD)** | **Post M**  **(SD)** | **Cohen’s *d* with CI** | **CLES** | ***n*** | ***p*** |
| **State decentering** | | 16.14  (5.34) | 17.45  (5.43) | 0.24 [-0.01, 0.50] | 0.57% | 117 | 0.025 |
| **State mindfulness** | | 72.32  (14.06) | 77.01  (13.21) | 0.34 [0.09, 0.60] | 0.60% | 117 | < 0.001 |

This table depicts the within-group analyses (whole sample) for state-level effects pre-post two weeks of mindfulness training using a complete-case approach. State mindfulness (SMS) and decentering (TMS) scores were reported after the first mindfulness session (T2; ‘Pre’) and after the second mindfulness session (T4; ‘Post’). The non-parametric test for examining within-group pre-post changes (Wilcoxon Signed-Rank) was used and effect sizes with confidence intervals for effect size were reported using Cohen’s *d* along with the adjusted calculation using common language effect size (CLES), using an online calculator to transform Cohen’s *d* to CLES: https://www.psychometrica.de/effect_size.html. Cohen’s *d* conventions for small (0.2), medium (0.5), and large effects (0.8) were used for interpretation.*Cases were excluded if participants indicated that they experienced no body sensations, emotions, or thoughts. For state mindfulness, there was one case missing from the pre-scores (*n* = 117). For state decentering, there was also one case missing from the pre-scores (*n* = 117).

1. **Between-group effects**

**Between-group analyses for state-level variables**

| **Variables** | **Time points** | **Degrees of freedom (df)** | **Test statistic**  **(*Quade’s)*** | **p values** | **Partial eta squared with CI** |
| --- | --- | --- | --- | --- | --- |
| **First mindfulness session** | | | | |  |
| State self-compassion | *T2* | (3, 127) | 1.80 | 0.151 | 0.04  [0.00, 0.09] |
| Pleasantness of thoughts | *T2* | (3, 127) | 1.42 | 0.240 | 0.03  [0.00, 0.08] |
| Pleasantness of emotions | *T2* | (3, 124) | 0.65 | 0.582 | 0.02  [0.00, 0.05] |
| Pleasantness of body sensations | *T2* | (3, 126) | 0.92 | 0.433 | 0.02  [0.00, 0.06] |
| **Second mindfulness session** | | | | |  |
| State self-compassion | *T4* | (3, 114) | 0.64 | 0.589 | 0.02  [0.00, 0.05] |
| Pleasantness of thoughts | *T4* | (3, 114) | 0.88 | 0.454 | 0.02  [0.00, 0.06] |
| Pleasantness of emotions | *T4* | (3, 112) | 1.75 | 0.161 | 0.05  [0.00, 0.10] |
| Pleasantness of body sensations | *T4* | (3, 112) | 3.52 | 0.017 | 0.09  [0.01, 0.16] |
| State mindfulness | *T4* | (3, 113) | 1.08 | 0.359 | 0.03  [0.00, 0.07] |
| State decentering | *T4* | (3, 113) | 0.05 | 0.984 | 0.001  [0.000, 0.000] |

This table depicts the non-parametric ANCOVA (Quade’s) test within the whole sample (Study 1) using a complete-case approach for between-group differences in post-scores (T2, T4) whilst controlling for pre-scores (T1, T3). The time points, degrees of freedom, test statistics, and significance values are reported. Pre-post scores are first ranked and then the one-way ANOVA test is run using the unstandardized residuals of the post-scores by the pre-scores to retrieve the degrees of freedom, test statistic, and p-values for the non-parametric ANCOVA (Quade’s) test using SPSS. Partial eta squared, as an estimate for effect size, [0.01 = small, 0.06 = medium, 0.14 = large] was calculated running a univariate general linear regression using the residuals of the post-scores controlling for the pre-scores and using the group variable as the fixed factor. The confidence intervals (CIs) for partial eta squared were estimated using SPSS script adapted by Kline (2004).

**Between-group analyses for state-level variables controlling for previous level of mindfulness experience, perceived impact of the Covid-19 pandemic, and format [online versus in-person]**

| **Variables** | **Time points** | **Degrees of freedom (df)** | **Test statistic**  **(*Quade’s)*** | ***p* values** | **Partial eta squared with CI** |
| --- | --- | --- | --- | --- | --- |
| **First mindfulness session** | | | | |  |
| State self-compassion | *T2* | (3, 107) | 1.99 | 0.121 | 0.05  [0.00, 0.11] |
| Pleasantness of thoughts | *T2* | (3, 107) | 1.14 | 0.335 | 0.03  [0.00, 0.08] |
| Pleasantness of emotions | *T2* | (3, 104) | 0.23 | 0.878 | 0.01  [0.00, 0.02] |
| Pleasantness of body sensations | *T2* | (3, 106) | 1.18 | 0.320 | 0.03  [0.00, 0.08] |
| **Second mindfulness session** | | | | |  |
| State self-compassion | *T4* | (3, 102) | 1.22 | 0.306 | 0.04  [0.00, 0.09] |
| Pleasantness of thoughts | *T4* | (3, 102) | 0.79 | 0.500 | 0.02  [0.00, 0.06] |
| Pleasantness of emotions | *T4* | (3, 100) | 1.70 | 0.173 | 0.05  [0.00, 0.11] |
| Pleasantness of body sensations | *T4* | (3, 100) | 2.70 | 0.050 | 0.08  [0.00, 0.15] |
| State mindfulness | *T4* | (3, 101) | 0.55 | 0.647 | 0.02  [0.00, 0.05] |
| State decentering | *T4* | (3, 101) | 0.08 | 0.973 | 0.002  [0.000, 0.006] |

This table depicts the non-parametric ANCOVA (Quade’s) test within the whole sample (Study 1) using a complete-case approach for between-group differences in post-scores (T2, T4) whilst controlling for pre-scores (T1, T3) previous mindfulness experience [MBCT graduate versus MBP naïve], format [online versus in-person], and perceived impact of the Covid-19 pandemic as covariates. The time points, degrees of freedom, test statistics, and significance values are reported. The dependent variables (post-scores) and covariates (pre-scores, previous mindfulness experience, format, and perceived impact of the Covid-19 pandemic) are first ranked and then the one-way ANOVA test is run using the unstandardized residuals of the post-scores by the covariates to retrieve the degrees of freedom, test statistic, and p-values for the non-parametric ANCOVA (Quade’s) test using SPSS. Partial eta squared, as an estimate for effect size, [0.01 = small, 0.06 = medium, 0.14 = large] was calculated running a univariate general linear regression using the residuals of the post-scores controlling for the covariates and using the group variable as the fixed factor. The confidence intervals (CIs) for partial eta squared were estimated using SPSS script adapted by Kline (2004). Previous level of mindfulness experience [MBCT graduate versus MBP naïve] was established at T0 when participants were screened. In light of the Covid-19 pandemic, a sub-group of the sample completed the study online versus in-person. Retrospectively, after taking part in the study, participants were asked about the extent to which their participation in the study was impacted by the Covid-19 pandemic. Participants responded on a Likert scale from 0 (*Not at all*) to 3 (*Very much*). Participants were instructed to indicate *Not at all* if their participation in the study did not coincide with the Covid-19 pandemic. Therefore, the responses to this question were used to infer the proportion of those that took part online during the Covid-19 pandemic versus those that took part in-person before the Covid-19 pandemic. Those that indicated *Not at all* (*n* = 65) were assigned a 0 to infer participation in-person and those that indicated ‘a little’ or ‘a moderate amount’ or ‘very much’ (*n* = 46) were assigned a 1 to infer participation online. This dummy coding was used to infer the ‘format’ of delivery [online versus in-person]. Using this dummy coding, no meaningful differences was found in terms of ‘study format’ across the four experimental groups: (Body scan: 14 [[51.85%] face-to-face; Mindful movement: 17 [68.00%] face-to-face; Breath and body: 18 [62.07%] face-to-face; and Befriending: 15 [53.57%] face-to-face) [*p* = 0.612]. Based on the hypothesis that the Covid-19 pandemic would negatively affect mental health scores, we looked at the anxiety and depression scores at baseline across these groups. Looking at the data descriptively, we did not find any meaningful differences in terms of depression scores [online: *M* = 3.20, *SD* = 3.03, n = 45; face-to-face: *M* = 3.09, *SD* = 2.86, n = 64]. In terms of anxiety scores, the online cohort indicated slightly higher scores [*M* = 4.59, *SD* = 3.62, *n* = 44] compared to the face-to-face cohort [*M* = 3.33, *SD* = 2.96, *n* = 64], but remained in the none-to-minimal range and therefore was not clinically relevant. The responses to this question were also used to look at average levels of perceived impact (scores ranged from 0-3) to control for this variable as an additional covariate. Participants were retrospectively asked the question: ‘Did the Covid-19 pandemic affect you during the two-week study’ on a Likert scale of 0-3 (*Not at all* to *Very much*). Out of those included at baseline (*n* = 131), a sub-group provided data for this question (*n* = 111) [84.73% response rate]. Within the whole sample that provided data to this question, 49.60% (*n* = 65) reported that they were affected *Not at all*, 18.30% (*n* = 24) reported that they were affected ‘a little’, 9.20% (*n* = 12) reported that they were affected ‘a moderate amount’ and 7.60% (*n* = 10) reported that they were affected *very much*. Across the four experimental groups, no meaningful difference in perceived impact of the Covid-19 pandemic was demonstrated (Body scan: *M* = 0.79, *SD* = 1.03; Mindful movement: *M* = 0.52, *SD* = 0.87; Breath and body: *M* = 0.76, *SD* = 1.12; Befriending: *M* = 0.72, *SD* = 0.92; *p* = .716).’ When we looked at the anxiety scores at baseline in those that indicated *not at all* affected [*M* = 3.33, *SD* = 2.96, *n* = 64] versus *very much* affected [*M* = 5.30, *SD* = 5.46, *n* = 10], it looks like there is a meaningful difference with the *not at all* group in the none-to-minimal range but the *very much* group in the mild range. In terms of depression, it did not seem like there was a meaningful difference across the *not at all* [*M* = 3.09, *SD* = 2.86, *n* = 64] and *very much* affected groups [*M* = 3.10, *SD* = 3.78, *n* = 10]. Based on these differences in terms of anxiety scores, it makes sense to control for ‘perceived impact of the Covid-19 pandemic’ in our sensitivity analyses.

**Pre-post means (standard deviations) within each experimental group for the first mindfulness session [T1-T2] (Study 1)**

|  | **Body scan**  **( *n* = 32)** | | | **Mindful movement**  **(*n* = 32)** | | | **Breath and body**  **(*n* = 37)** | | | **Befriending**  **(*n* = 30)** | | |  |
| --- | --- | --- | --- | --- | --- | --- | --- | --- | --- | --- | --- | --- | --- |
|  | **Pre *M***  ***(SD)*** | **Post *M***  ***(SD)*** | ***n*** | **Pre *M***  ***(SD)*** | **Post *M***  ***(SD)*** | ***n*** | **Pre *M***  ***(SD)*** | **Post *M***  ***(SD)*** | ***n*** | **Pre *M***  ***(SD)*** | **Post *M***  ***(SD)*** | ***n*** | |
| **Perceived pleasantness of body sensations** | 46.50  (15.94) | 57.16  (19.33) | 32 | 45.39  (16.18) | 63.39  (17.39) | 31* | 40.89  (22.77) | 57.84  (17.29) | 37 | 45.30  (15.96) | 60.10  (13.51) | 30 | |
| **Perceived pleasantness of emotions** | 55.33  (14.63) | 60.70  (17.96) | 30* | 53.58  (20.09) | 65.65  (21.87) | 31* | 51.05  (17.87) | 61.81  (23.09) | 37 | 56.47  (18.37) | 60.03  (20.58) | 30 | |
| **Perceived pleasantness of thoughts** | 52.41  (16.29) | 56.53  (16.95) | 32 | 59.03  (16.22) | 64.38  (17.82) | 32 | 50.41  (20.52) | 62.54  (14.69) | 37 | 55.30  (14.48) | 58.90  (15.74) | 30 | |
| **State self-compassion** | 64.14  (20.68) | 70.11  (18.00) | 32 | 73.03  (16.15) | 78.00  (14.46) | 32 | 56.38  (21.49) | 67.58  (20.33) | 37 | 67.52  (15.73) | 76.87  (13.37) | 30 | |

This table depicts the pre-post means (standard deviations) and n for each experimental group for the first mindfulness session using a complete-case approach. *Cases were excluded if participants indicated that they experienced no body sensations, emotions, or thoughts.

**Pre-post means (standard deviations) within each experimental group for the second mindfulness session [T3-T4] (Study 1)**

|  | **Body scan**  **(*n* = 31)** | | | **Mindful movement**  **(*n* = 29)** | | | **Breath and body**  **(*n* = 31)** | | | **Befriending**  **(*n* = 27)** | | |  |
| --- | --- | --- | --- | --- | --- | --- | --- | --- | --- | --- | --- | --- | --- |
|  | **Pre *M***  ***(SD)*** | **Post *M***  ***(SD)*** | ***n*** | **Pre *M***  ***(SD)*** | **Post *M***  ***(SD)*** | ***n*** | **Pre *M***  ***(SD)*** | **Post *M***  ***(SD)*** | ***n*** | **Pre *M***  ***(SD)*** | **Post *M***  ***(SD)*** | ***n*** | |
| **Perceived pleasantness of body sensations** | 56.26  (23.02) | 62.84  (20.54) | 31 | 48.46  (18.75) | 67.24  (20.37) | 28* | 51.43  (20.34) | 58.55  (23.52) | 30* | 48.70  (18.05) | 55.81  (20.04) | 27 | |
| **Perceived pleasantness of emotions** | 54.50  (14.48) | 54.29  (16.22) | 30* | 59.14  (21.26) | 67.69  (21.81) | 28* | 52.87  (18.27) | 63.58  (18.25) | 31 | 54.22  (20.90) | 62.26  (12.35) | 27 | |
| **Perceived pleasantness of thoughts** | 54.26  (17.59) | 55.35  (16.92) | 31 | 59.21  (18.36) | 63.52  (22.48) | 29 | 50.58  (12.67) | 58.90  (18.26) | 31 | 49.70  (19.46) | 56.07  (18.45) | 27 | |
| **State self-compassion** | 69.18  (14.44) | 73.44  (14.49) | 31 | 80.36  (11.76) | 83.71  (10.46) | 29 | 65.82  (15.12) | 72.45  (17.22) | 31 | 71.28  (17.30) | 77.87  (17.22) | 27 | |

This table depicts the pre-post means (standard deviations) and n for each experimental group for the second mindfulness session using a complete-case approach. *Cases were excluded if participants indicated that they experienced no body sensations, emotions, or thoughts.

**Pre-post means (standard deviations) within each experimental group pre-post two weeks of daily mindfulness practice [T2-T4] (Study 1)**

|  | **Body scan**  **(*n* = 31)** | | | **Mindful movement**  **(*n* = 29)** | | | **Breath and body**  **(*n* = 31)** | | | **Befriending**  **(*n* = 26)** | | |  |
| --- | --- | --- | --- | --- | --- | --- | --- | --- | --- | --- | --- | --- | --- |
|  | **Pre *M***  ***(SD)*** | **Post *M***  ***(SD)*** | ***n*** | **Pre *M***  ***(SD)*** | **Post *M***  ***(SD)*** | ***n*** | **Pre *M***  ***(SD)*** | **Post *M***  ***(SD)*** | ***n*** | **Pre *M***  ***(SD)*** | **Post *M***  ***(SD)*** | ***n*** | |
| **State decentering** | 14.52  (5.06) | 16.26  (5.99) | 31 | 16.93  (5.69) | 18.31  (4.90) | 29 | 16.35  (5.95) | 17.29  (5.98) | 31 | 16.92  (4.24) | 17.88  (4.61) | 26 | |
| **State mindfulness** | 69.55  (13.98) | 75.90  (12.80) | 31 | 73.45  (16.49) | 79.59  (11.86) | 29 | 72.97  (12.63) | 74.39  (14.49) | 31 | 73.58  (13.18) | 77.81  (13.27) | 26 | |

This table depicts the pre-post means (standard deviations) and n for each experimental group pre-post two weeks of daily mindfulness practice using a complete-case approach. *Cases were excluded if participants indicated that they experienced no body sensations, emotions, or thoughts.

1. **The strong association criterion – Study 1**

**Associations between change in candidate mechanisms and change in outcomes within the whole sample (Study 1)**

| **Mediator**  (Differential scores) | **Outcome**  (Differential scores) | **Time point** | **Output** |
| --- | --- | --- | --- |
| State self-compassion | Pleasantness of body sensations | T1-T2 | r _(127)_ = 0.22 [0.05, 0.38], p = 0.011 |
| State self-compassion | Pleasantness of thoughts | T1-T2 | r _(128)_ = 0.10 [-0.07, 0.27], p = 0.256 |
| State self-compassion | Pleasantness of emotions | T1-T2 | r _(125)_ = 0.16 [-0.02, 0.33], p = 0.067 |
| State self-compassion | Pleasantness of body sensations | T3-T4 | r _(113)_ = 0.28 [0.10, 0.44], p = 0.002 |
| State self-compassion | Pleasantness of thoughts | T3-T4 | r _(115)_ = 0.19 [0.01, 0.36], p = 0.043 |
| State self-compassion | Pleasantness of emotions | T3-T4 | r _(113)_ = 0.29 [0.11, 0.45], p = 0.002 |
| State mindfulness | Pleasantness of body sensations | T2-T4 | r _(113)_ = 0.22 [0.04, 0.39], p = 0.019 |
| State mindfulness | Pleasantness of thoughts | T2-T4 | r _(114)_ = 0.21 [0.03, 0.38], p = 0.024 |
| State mindfulness | Pleasantness of emotions | T2-T4 | r _(111)_ = 0.13 [-0.06, 0.31], p = 0.193 |
| State decentering | Pleasantness of body sensations | T2-T4 | r _(113)_ = 0.11 [-0.08, 0.29], p = 0.234 |
| State decentering | Pleasantness of thoughts | T2-T4 | r _(114)_ = 0.08 [-0.10, 0.26], p = 0.389 |
| State decentering | Pleasantness of emotions | T2-T4 | r _(111)_ = 0.09 [-0.10, 0.27], p = 0.357 |

This table depicts the non-parametric partial rank correlations within the whole sample (Study 1) for the associations between the differences in scores for candidate mechanisms and the differences in scores for proposed outcomes whilst controlling for baseline levels in outcomes. Partial rank correlations were calculated (Conover, 1999). Estimates for the confidence intervals for the r-values were calculated using this online calculator here: <http://vassarstats.net/rho.html>. The size of the associations (r-values) were interpreted using the conventions outlined by Hattie, J. (2011) [0.00-0.10 = no effect, 0.10-0.20= small effect, 0.20-0.30 = moderate effect, r > 0.30 = large effect] (Hattie, 2011).

**Associations between change in proposed mediators and change in outcomes within the body scan group (Study 1)**

| **Mediator**  (Differential scores) | **Outcome**  (Differential scores) | **Time point** | **Output** |
| --- | --- | --- | --- |
| State self-compassion | Pleasantness of body sensations | T1-T2 | r _(29)_ = 0.47 [0.13, 0.71], p = 0.007 |
| State self-compassion | Pleasantness of thoughts | T1-T2 | r _(29)_ = 0.10 [-0.27, 0.44], p = 0.590 |
| State self-compassion | Pleasantness of emotions | T1-T2 | r _(27)_ = 0.19 [-0.20, 0.53], p = 0.315 |
| State self-compassion | Pleasantness of body sensations | T3-T4 | r _(28)_ = 0.40 [0.04, 0.67], p =0.030 |
| State self-compassion | Pleasantness of thoughts | T3-T4 | r _(28)_ = 0.28 [-0.10, 0.59], p = 0.139 |
| State self-compassion | Pleasantness of emotions | T3-T4 | r _(27)_ = 0.28 [-0.10, 0.59], p = 0.136 |
| State mindfulness | Pleasantness of body sensations | T2-T4 | r _(28)_ = -0.05 [-0.41, 0.32], p = 0.813 |
| State mindfulness | Pleasantness of thoughts | T2-T4 | r _(28)_ = 0.06 [-0.31, 0.42], p = 0.735 |
| State mindfulness | Pleasantness of emotions | T2-T4 | r _(26)_ = 0.02 [-0.36, 0.40], p = 0.908 |
| State decentering | Pleasantness of body sensations | T2-T4 | r _(28)_ = 0.11 [-0.27, 0.46], p = 0.547 |
| State decentering | Pleasantness of thoughts | T2-T4 | r _(28)_ = 0.21 [-0.17, 0.54], p = 0.277 |
| State decentering | Pleasantness of emotions | T2-T4 | r _(26)_ = 0.40 [0.02, 0.68], p = 0.035 |

This table depicts the non-parametric partial rank correlations within the body scan group only (Study 1) for the associations between the differences in scores for candidate mechanisms and the differences in scores for proposed outcomes whilst controlling for baseline levels in outcomes. Partial rank correlations were calculated (Conover, 1999). Estimates for the confidence intervals for the r-values were calculated using this online calculator here: <http://vassarstats.net/rho.html>. The size of the associations (r-values) were interpreted using the conventions outlined by Hattie, J. (2011) [0.00-0.10 = no effect, 0.10-0.20= small effect, 0.20-0.30 = moderate effect, r > 0.30 = large effect] (Hattie, 2011).

**Associations between change in proposed mediators and change in outcomes within the mindful movement group (Study 1)**

| **Mediator**  (Differential scores) | **Outcome**  (Differential scores) | **Time point** | **Output** |
| --- | --- | --- | --- |
| State self-compassion | Pleasantness of body sensations | T1-T2 | r _(28)_ = 0.02 [-0.35, 0.38], p = 0.922 |
| State self-compassion | Pleasantness of thoughts | T1-T2 | r _(29)_ = -0.04 [-0.39, 0.32], p = 0.839 |
| State self-compassion | Pleasantness of emotions | T1-T2 | r _(28)_ = 0.02 [-0.35, 0.38], p = 0.912 |
| State self-compassion | Pleasantness of body sensations | T3-T4 | r _(25)_ = 0.09 [-0.31, 0.46], p = 0.657 |
| State self-compassion | Pleasantness of thoughts | T3-T4 | r _(26)_ = 0.27 [-0.12, 0.59], p = 0.168 |
| State self-compassion | Pleasantness of emotions | T3-T4 | r _(25)_ = 0.24 [-0.16, 0.57], p = 0.223 |
| State mindfulness | Pleasantness of body sensations | T2-T4 | r _(25)_ = 0.29 [-0.11, 0.61], p = 0.147 |
| State mindfulness | Pleasantness of thoughts | T2-T4 | r _(26)_ = 0.29 [-0.10, 0.60], p = 0.139 |
| State mindfulness | Pleasantness of emotions | T2-T4 | r _(25)_ = -0.20 [-0.55, 0.20], p = 0.319 |
| State decentering | Pleasantness of body sensations | T2-T4 | r _(25)_ = 0.39 [0.00, 0.68], p = 0.043 |
| State decentering | Pleasantness of thoughts | T2-T4 | r _(26)_ = 0.19 [-0.20, 0.53], p = 0.321 |
| State decentering | Pleasantness of emotions | T2-T4 | r _(25)_ = -0.10 [-0.47, 0.30], p = 0.604 |

This table depicts the non-parametric partial rank correlations within the mindful movement group only (Study 1) for the associations between the differences in scores for candidate mechanisms and the differences in scores for proposed outcomes whilst controlling for baseline levels in outcomes. Partial rank correlations were calculated (Conover, 1999). Estimates for the confidence intervals for the r-values were calculated using this online calculator here: <http://vassarstats.net/rho.html>. The size of the associations (r-values) were interpreted using the conventions outlined by Hattie, J. (2011) [0.00-0.10 = no effect, 0.10-0.20= small effect, 0.20-0.30 = moderate effect, r > 0.30 = large effect] (Hattie, 2011).

**Associations between change in proposed mediators and change in outcomes within the breath and body group (Study 1)**

| **Mediator**  (Differential scores) | **Outcome**  (Differential scores) | **Time point** | **Output** |
| --- | --- | --- | --- |
| State self-compassion | Pleasantness of body sensations | T1-T2 | r _(34)_ = 0.15 [-0.19, 0.46], p = 0.370 |
| State self-compassion | Pleasantness of thoughts | T1-T2 | r _(34)_ = 0.12 [-0.22, 0.44], p = 0.491 |
| State self-compassion | Pleasantness of emotions | T1-T2 | r _(34)_ = 0.10 [-0.24, 0.42], p = 0.546 |
| State self-compassion | Pleasantness of body sensations | T3-T4 | r _(27)_ = 0.30 [-0.08, 0.61], p = 0.120 |
| State self-compassion | Pleasantness of thoughts | T3-T4 | r _(28)_ =0.04 [-0.33, 0.40], p = 0.854 |
| State self-compassion | Pleasantness of emotions | T3-T4 | r _(28)_ = 0.32 [-0.05, 0.61], p = 0.082 |
| State mindfulness | Pleasantness of body sensations | T2-T4 | r _(28)_ =0.05 [-0.32, 0.41], p = 0.812 |
| State mindfulness | Pleasantness of thoughts | T2-T4 | r _(28)_ = 0.17 [-0.21, 0.51], p = 0.380 |
| State mindfulness | Pleasantness of emotions | T2-T4 | r _(28)_ = 0.39 [0.03, 0.66], p = 0.033 |
| State decentering | Pleasantness of body sensations | T2-T4 | r _(28)_ = 0.10 [-0.28, 0.45], p = 0.604 |
| State decentering | Pleasantness of thoughts | T2-T4 | r _(28)_ = 0.07 [-0.30, 0.43], p = 0.698 |
| State decentering | Pleasantness of emotions | T2-T4 | r _(28)_ = 0.29 [-0.09, 0.59], p = 0.126 |

This table depicts the non-parametric partial rank correlations within the breath and body group only (Study 1) for the associations between the differences in scores for candidate mechanisms and the differences in scores for proposed outcomes whilst controlling for baseline levels in outcomes. Partial rank correlations were calculated (Conover, 1999). Estimates for the confidence intervals for the r-values were calculated using this online calculator here: <http://vassarstats.net/rho.html>. The size of the associations (r-values) were interpreted using the conventions outlined by Hattie, J. (2011) [0.00-0.10 = no effect, 0.10-0.20= small effect, 0.20-0.30 = moderate effect, r > 0.30 = large effect] (Hattie, 2011).

**Associations between change in proposed mediators and change in outcomes within the befriending group (Study 1)**

| **Mediator**  (Differential scores) | **Outcome**  (Differential scores) | **Time point** | **Output** |
| --- | --- | --- | --- |
| State self-compassion | Pleasantness of body sensations | T1-T2 | r _(27)_ = 0.08 [-0.30, 0.44], p = 0.679 |
| State self-compassion | Pleasantness of thoughts | T1-T2 | r _(27)_ = 0.22 [-0.17, 0.55], p = 0.260 |
| State self-compassion | Pleasantness of emotions | T1-T2 | r _(27)_ = 0.31 [-0.07, 0.61], p = 0.104 |
| State self-compassion | Pleasantness of body sensations | T3-T4 | r _(24)_ = 0.29 [-0.12, 0.61], p = 0.146 |
| State self-compassion | Pleasantness of thoughts | T3-T4 | r _(24)_ = 0.28 [-0.13, 0.61], p = 0.161 |
| State self-compassion | Pleasantness of emotions | T3-T4 | r _(24)_ = 0.23 [-0.18, 0.57], p = 0.263 |
| State mindfulness | Pleasantness of body sensations | T2-T4 | r _(23)_ = 0.42 [0.02, 0.70], p = 0.038 |
| State mindfulness | Pleasantness of thoughts | T2-T4 | r _(23)_ = 0.19 [-0.23, 0.55], p = 0.354 |
| State mindfulness | Pleasantness of emotions | T2-T4 | r _(23)_ = 0.33 [-0.08, 0.65], p = 0.112 |
| State decentering | Pleasantness of body sensations | T2-T4 | r _(23)_ = -0.18 [-0.54, 0.24], p = 0.387 |
| State decentering | Pleasantness of thoughts | T2-T4 | r _(23)_ = -0.52 [-0.76, -0.15], p = 0.007 |
| State decentering | Pleasantness of emotions | T2-T4 | r _(23)_ = -0.02 [-0.42, 0.39], p = 0.922 |

This table depicts the non-parametric partial rank correlations within the befriending group only (Study 1) for the associations between the differences in scores for candidate mechanisms and the differences in scores for proposed outcomes whilst controlling for baseline levels in outcomes. Partial rank correlations were calculated (Conover, 1999). Estimates for the confidence intervals for the r-values were calculated using this online calculator here: <http://vassarstats.net/rho.html>. The size of the associations (r-values) were interpreted using the conventions outlined by Hattie, J. (2011) [0.00-0.10 = no effect, 0.10-0.20= small effect, 0.20-0.30 = moderate effect, r > 0.30 = large effect] (Hattie, 2011).

1. **Logic Diagram for Study 1**


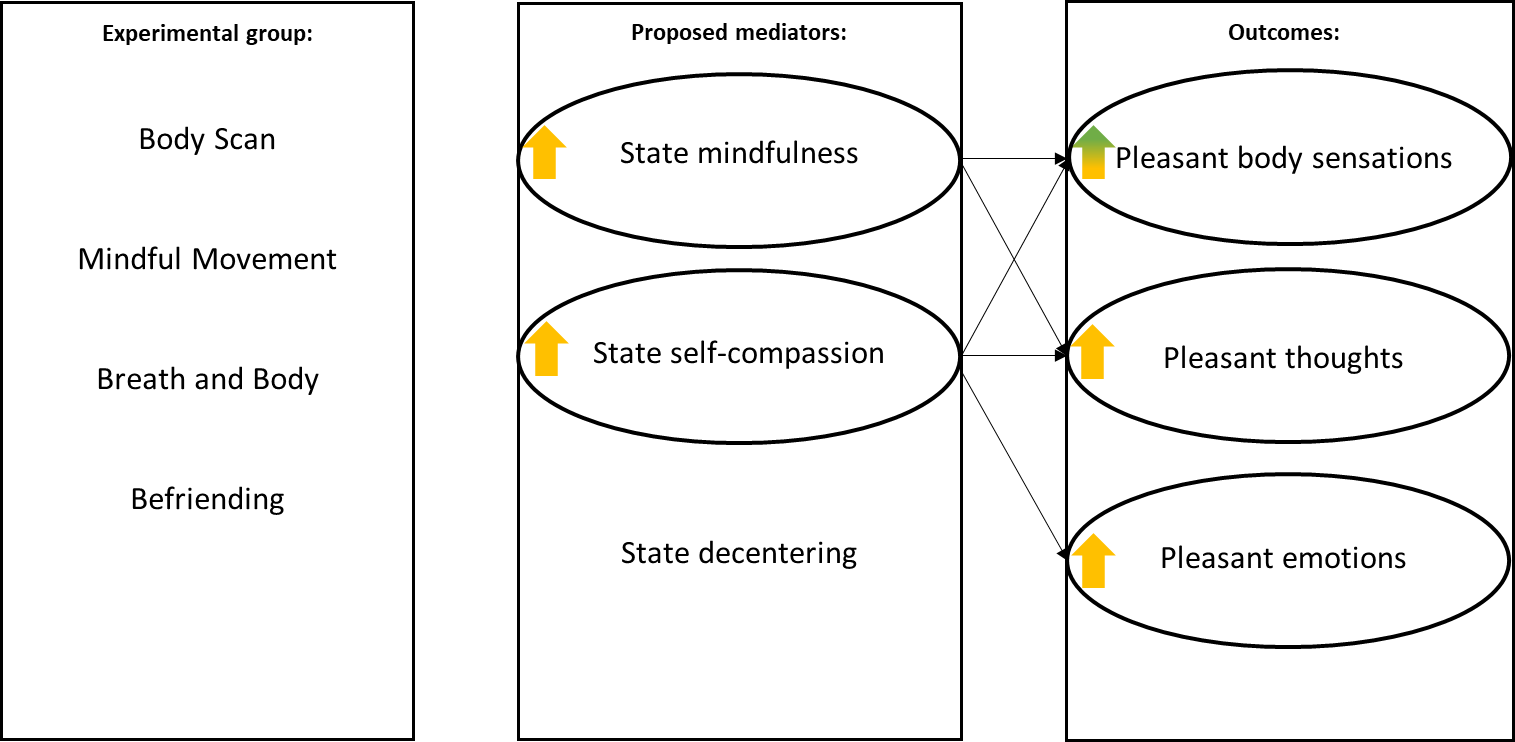


This logic diagram illustrates the key hypotheses that were generated from Study 1. The large arrows are used to indicate the within-group effects within the whole sample for candidate mechanisms and outcomes (e.g., increase in state self-compassion and pleasant body sensations). The yellow arrows are used to indicate small to medium effects whereas the green arrows are used to indicate medium to large effects. For pleasant body sensations, the arrow is indicated as yellow to green given that Study 1 found small to large effects across multiple time-points for this outcome. The solid black lines with arrows indicate the small to moderate correlations found within the whole sample between changes in candidate mechanisms and outcomes. The aim of this diagram is to help guide future research. Please note that analyses within the whole sample were prioritized due to larger sample size and therefore not all correlations are reported.

1. **Study 2 results**
2. **Within-group effects**

**Within-group analyses (whole sample) for trait-level effects pre-post two weeks of daily mindfulness training (Study 2)**

|  |  | **Whole sample (*n*=121)** | | | | |
| --- | --- | --- | --- | --- | --- | --- |
|  | **Pre *M***  ***(SD)*** | | **Post *M***  ***(SD)*** | **Cohen’s *d* with CI** | **CLES** | ***p*** |
| **Psychological quality of life** | 64.81  (13.26) | | 68.29  (13.19) | 0.26 [0.01, 0.52] | 0.57% | < 0.001 |
| **Anxiety*** | 3.71  (3.19) | | 4.85  (3.90) | 0.32 [0.07, 0.57] | 0.59% | 0.001 |
| **Depression** | 3.14  (2.90) | | 3.45  (2.58) | 0.11 [-0.14, 0.37] | 0.53% | 0.155 |
| **Trait self-compassion** | 3.36  (0.71) | | 3.56  (0.68) | 0.29 [0.03, 0.54] | 0.58% | < 0.001 |
| **Trait decentering** | 3.30  (0.56) | | 3.63  (0.50) | 0.62 [0.36, 0.88] | 0.67% | <0.001 |
| **Attentional control** | 30.00  (5.84) | | 31.97  (6.31) | 0.32 [0.07, 0.58] | 0.59% | < 0.001 |
| **Trait mindfulness** | 40.28  (6.55) | | 42.37  (6.33) | 0.32 [0.07, 0.58] | 0.59% | < 0.001 |
| **Observing** | 9.94  (2.48) | | 11.05  (2.32) | 0.46 [0.21, 0.72] | 0.63% | < 0.001 |
| **Describing** | 10.83  (2.45) | | 11.02  (2.36) | 0.08 [-0.17, 0.33] | 0.52% | 0.281 |
| **Acting with awareness** | 9.08  (2.33) | | 9.51  (2.34) | 0.18 [-0.07, 0.44] | 0.55% | 0.009 |
| **Non-judgement** | 11.23  (2.75) | | 11.78  (2.55) | 0.21 [-0.05, 0.46] | 0.56% | 0.028 |
| **Non-reactivity** | 9.13  (2.35) | | 10.06  (2.20) | 0.41 [0.15, 0.66] | 0.61% | < 0.001 |
| **Noticing** | 3.03  (1.05) | | 3.46  (.81) | 0.46 [0.20, 0.71] | 0.63% | < 0.001 |
| **Attentional regulation** | 2.53  (1.08) | | 3.11  (.96) | 0.57 [0.31, 0.83] | 0.66% | < 0.001 |
| **Trusting** | 3.26  (1.14) | | 3.72  (1.00) | 0.43 [0.17, 0.68] | 0.62% | < 0.001 |
| **Body listening** | 2.25  (1.23) | | 3.03  (1.20) | 0.64 [0.38, 0.90] | 0.68% | < 0.001 |
| **Emotional awareness** | 3.15  (1.17) | | 3.72  (1.00) | 0.52 [0.27, 0.78] | 0.64% | < 0.001 |

This table depicts the within-group analyses (whole sample) for trait-level effects pre-post two weeks of training using a complete-case approach. Psychological quality of life (WHO-BREF-QOL [Psychological Domain]), anxiety (GAD-7), depression (PHQ-9), self-compassion (SCS-SF), decentring (EQ), attentional control (ACS-SF), mindfulness (FFMQ-15), observing, describing, acting with awareness, non-judgement, non-reactivity (subscales of FFMQ-15), noticing, attention regulation, trusting, body listening, and emotional awareness (subscales of MAIA-2) scores are reported before two weeks of daily training (T1; ‘Pre’) and after two weeks of daily mindfulness training (T4; ‘Post’). The non-parametric test for examining within-group pre-post changes (Wilcoxon Signed-Rank) was used and effect sizes with confidence intervals for effect size were reported using Cohen’s *d* along with the adjusted calculation using common language effect size (CLES), using an online calculator to transform Cohen’s *d* to CLES: https://www.psychometrica.de/effect_size.html. Cohen’s *d* conventions for small (0.2), medium (0.5), and large effects (0.8) were used for interpretation*One case was missing for the anxiety measure at pre-intervention (*n* = 120).

1. **Between-group effects**

**Between-group analyses for trait-level variables**

| **Variable** | **Time points** | **Degree of freedom (df)** | **Test statistic (*Quade’s*)** | ***p* values** | **Partial eta squared with CI** |
| --- | --- | --- | --- | --- | --- |
| Psychological quality of life | *T4* | (3, 117) | 0.53 | 0.663 | 0.01  [0.00, 0.04] |
| Anxiety | *T4* | (3, 116) | 1.22 | 0.304 | 0.03  [0.00, 0.08] |
| Depression | *T4* | (3, 117) | 2.50 | 0.063 | 0.06  [0.00, 0.12] |
| Trait self-compassion | *T4* | (3, 117) | 1.35 | 0.262 | 0.03  [0.00, 0.08] |
| Trait decentering | *T4* | (3, 117) | 1.59 | 0.196 | 0.04  [0.00, 0.10] |
| Attentional control | *T4* | (3, 117) | 0.37 | 0.774 | 0.01  [0.00, 0.03] |
| Trait mindfulness | *T4* | (3, 117) | 0.57 | 0.637 | 0.01  [0.00, 0.04] |
| Observing | *T4* | (3, 117) | 0.71 | 0.547 | 0.02  [0.00, 0.05] |
| Describing | *T4* | (3, 117) | 1.22 | 0.307 | 0.03  [0.00, 0.08] |
| Acting with awareness | *T4* | (3, 117) | 0.90 | 0.444 | 0.02  [0.00, 0.06] |
| Non-judgement | *T4* | (3, 117) | 1.43 | 0.238 | 0.04  [0.00, 0.08] |
| Non-reactivity | *T4* | (3, 117) | 0.81 | 0.489 | 0.02  [0.00, 0.06] |
| Noticing | *T4* | (3, 117) | 0.84 | 0.477 | 0.02  [0.00, 0.06] |
| Attentional regulation | *T4* | (3, 117) | 0.28 | 0.840 | 0.01  [0.00, 0.02] |
| Trusting | *T4* | (3, 117) | 1.14 | 0.335 | 0.03  [0.00, 0.07] |
| Body listening | *T4* | (3, 117) | 0.49 | 0.690 | 0.01  [0.00, 0.04] |
| Emotional awareness | *T4* | (3, 117) | 0.19 | 0.901 | 0.01  [0.00, 0.01] |

This table depicts the non-parametric ANCOVA (Quade’s) test within the whole sample (Study 2) using a complete-case approach for between-group differences in post-intervention scores (T4) whilst controlling for baseline (T1). The time points, degrees of freedom, test statistics, and the significance values are reported. Pre-post scores are first ranked and then the one-way ANOVA test is run using the unstandardized residuals of the post-scores by the pre-scores to retrieve the degrees of freedom, test statistic, and p-values for the non-parametric ANCOVA (Quade’s) test using SPSS. Partial eta squared, as an estimate for effect size, [0.01 = small, 0.06 = medium, 0.14 = large] was calculated running a univariate general linear regression using the residuals of the post-scores controlling for the pre-scores. The confidence intervals (CIs) for partial eta squared were estimated using SPSS script adapted by Kline (2004).

**Between-group analyses for state-level variables controlling for previous level of mindfulness experience, perceived impact of the Covid-19 pandemic, and format [online versus in-person]**

| **Variable** | **Time points** | **Degree of freedom (df)** | **Test statistic (*Quade’s*)** | ***p* values** | **Partial eta squared with CI** |
| --- | --- | --- | --- | --- | --- |
| Psychological quality of life | *T4* | (3, 105) | 1.59 | 0.195 | 0.04  [0.00, 0.10] |
| Anxiety | *T4* | (3, 104) | 1.82 | 0.149 | 0.05  [0.00, 0.11] |
| Depression | *T4* | (3, 105) | 2.32 | 0.080 | 0.06  [0.00, 0.13] |
| Trait self-compassion | *T4* | (3, 105) | 1.51 | 0.218 | 0.04  [0.00, 0.10] |
| Trait decentering | *T4* | (3, 105) | 1.90 | 0.135 | 0.05  [0.00, 0.11] |
| Attentional control | *T4* | (3, 105) | 0.36 | 0.786 | 0.01  [0.00, 0.03] |
| Trait mindfulness | *T4* | (3, 105) | 0.80 | 0.495 | 0.02  [0.00, 0.06] |
| Observing | *T4* | (3, 105) | 0.66 | 0.581 | 0.02  [0.00, 0.05] |
| Describing | *T4* | (3, 105) | 1.10 | 0.355 | 0.03  [0.00, 0.08] |
| Acting with awareness | *T4* | (3, 105) | 0.78 | 0.506 | 0.02  [0.00, 0.06] |
| Non-judgement | *T4* | (3, 105) | 1.88 | 0.138 | 0.05  [0.00, 0.11] |
| Non-reactivity | *T4* | (3, 105) | 0.46 | 0.710 | 0.01  [0.00, 0.04] |
| Noticing | *T4* | (3, 105) | 1.16 | 0.329 | 0.03  [0.00, 0.08] |
| Attentional regulation | *T4* | (3, 105) | 0.33 | 0.805 | 0.01  [0.00, 0.03] |
| Trusting | *T4* | (3, 105) | 1.53 | 0.210 | 0.04  [0.00, 0.10] |
| Body listening | *T4* | (3, 105) | 0.92 | 0.435 | 0.03  [0.00, 0.07] |
| Emotional awareness | *T4* | (3, 105) | 0.56 | 0.641 | 0.02  [0.00, 0.05] |

This table depicts the non-parametric ANCOVA (Quade’s) test within the whole sample (Study 2) using a complete-case approach for between-group differences in post-scores (T2, T4) whilst controlling for pre-scores (T1, T3) previous mindfulness experience [MBCT graduate versus MBP naïve], format [online versus in-person], and perceived impact of the Covid-19 pandemic as covariates. The time points, degrees of freedom, test statistics, and significance values are reported. The dependent variables (post-scores) and covariates (pre-scores, previous mindfulness experience, format, and perceived impact of the Covid-19 pandemic) are first ranked and then the one-way ANOVA test is run using the unstandardized residuals of the post-scores by the covariates to retrieve the degrees of freedom, test statistic, and p-values for the non-parametric ANCOVA (Quade’s) test using SPSS. Partial eta squared, as an estimate for effect size, [0.01 = small, 0.06 = medium, 0.14 = large] was calculated running a univariate general linear regression using the residuals of the post-scores controlling for the covariates and using the group variable as the fixed factor. The confidence intervals (CIs) for partial eta squared were estimated using SPSS script adapted by Kline (2004). Previous level of mindfulness experience [MBCT graduate versus MBP naïve] was established at T0 when participants were screened. In light of the Covid-19 pandemic, a sub-group of the sample completed the study online versus in-person. Retrospectively, after taking part in the study, participants were asked about the extent to which their participation in the study was impacted by the Covid-19 pandemic. Participants responded on a Likert scale from 0 (*Not at all*) to 3 (*Very much*). Participants were instructed to indicate *Not at all* if their participation in the study did not coincide with the Covid-19 pandemic. Therefore, the responses to this question were used to infer the proportion of those that took part online during the Covid-19 pandemic versus those that took part in-person before the Covid-19 pandemic. Those that indicated *Not at all* (*n* = 65) were assigned a 0 to infer participation in-person and those that indicated *a little* or *a moderate amount* or *very much* (*n* = 46) were assigned a 1 to infer participation online. This dummy coding was used to infer the ‘format’ of delivery [online versus in-person]. Using this dummy coding, no meaningful differences was found in terms of ‘study format’ across the four experimental groups: (Body scan: 14 [[51.85%] face-to-face; Mindful movement: 17 [68.00%] face-to-face; Breath and body: 18 [62.07%] face-to-face; and Befriending: 15 [53.57%] face-to-face) [p = .612]. Based on the hypothesis that the Covid-19 pandemic would negatively affect mental health scores, we looked at the anxiety and depression scores at baseline across these groups. Looking at the data descriptively, we did not find any meaningful differences in terms of depression scores [online: *M* = 3.20, *SD* = 3.03, *n* = 45; face-to-face: *M* = 3.09, *SD* = 2.86, *n* = 64]. In terms of anxiety scores, the online cohort indicated slightly higher scores [*M* = 4.59, *SD* = 3.62, *n* = 44] compared to the face-to-face cohort [*M* = 3.33, *SD* = 2.96, *n* = 64], but remained in the none-to-minimal range and therefore was not clinically relevant. The responses to this question were also used to look at average levels of perceived impact (scores ranged from 0-3) to control for this variable as an additional covariate. Participants were retrospectively asked the question: ‘Did the Covid-19 pandemic affect you during the two-week study’ on a Likert scale of 0-3 (*Not at all* to *Very much*). Out of those included at baseline (*n* = 131), a sub-group provided data for this question (*n* = 111) [84.73% response rate]. Within the whole sample that provided data to this question, 49.60% (*n* = 65) reported that they were affected *not at all*, 18.30% (*n* = 24) reported that they were affected *a little*, 9.20% (*n* = 12) reported that they were affected *a moderate amount* and 7.60% (*n* = 10) reported that they were affected *very much*. Across the four experimental groups, no meaningful difference in perceived impact of the Covid-19 pandemic was demonstrated (Body scan: *M* = 0.79, *SD* = 1.03; Mindful movement: *M* = 0.52, *SD* = 0.87; Breath and body: *M* = 0.76, *SD* = 1.12; Befriending: *M* = 0.72, *SD* = 0.92; *p* = .716).’ When we looked at the anxiety scores at baseline in those that indicated *not at all* affected [*M* = 3.33, *SD* = 2.96, *n* = 64] versus *very much* affected [*M* = 5.30, *SD* = 5.46, *n* = 10], it looks like there is a meaningful difference with the *not at all* group in the none-to-minimal range but the *very much* group in the mild range. In terms of depression, it did not seem like there was a meaningful difference across the *not at all* [*M* = 3.09, *SD* = 2.86, *n* = 64] and *very much* affected groups [*M* = 3.10, *SD* = 3.78, *n* = 10]. Based on these differences in terms of anxiety scores, it makes sense to control for ‘perceived impact of the Covid-19 pandemic’ in our sensitivity analyses.

**Pre-post means (standard deviations) within each experimental group pre-post two weeks of daily mindfulness practice [T1-T4] (Study 2)**

|  | **Body scan**  **(*n*=31)** | | **Mindful movement**  **(*n*=30)** | | **Breath and body**  **(*n*=31)** | | **Befriending**  **(*n*=29)** | |
| --- | --- | --- | --- | --- | --- | --- | --- | --- |
|  | **Pre *M***  ***(SD)*** | **Post *M***  ***(SD)*** | **Pre *M***  ***(SD)*** | **Post *M***  ***(SD)*** | **Pre *M***  ***(SD)*** | **Post *M***  ***(SD)*** | **Pre *M***  ***(SD)*** | **Post *M***  ***(SD)*** |
| **Psychological quality of life** | 64.65  (12.35) | 67.74  (12.59) | 67.08  (11.55) | 70.56  (11.16) | 60.75  (17.00) | 63.44  (15.05) | 66.95  (10.68) | 71.70  (12.67) |
| **Anxiety*** | 3.23  (2.96) | 5.00  (3.69) | 2.90  (3.08) | 3.77  (3.73) | 4.35  (3.41) | 5.42  (3.63) | 4.39  (3.14) | 5.21  (4.52) |
| **Depression** | 2.52  (2.43) | 3.16  (2.63) | 2.90  (2.93) | 2.60  (2.18) | 3.71  (3.24) | 4.55  (2.84) | 3.45  (2.94) | 3.48  (2.31) |
| **Trait self-compassion** | 3.39  (0.62) | 3.57  (0.54) | 3.53  (0.76) | 3.76  (0.72) | 3.09  (0.70) | 3.27  (0.67) | 3.43  (0.69) | 3.64  (0.72) |
| **Trait decentering** | 3.20  (0.54) | 3.49  (0.51) | 3.39  (0.69) | 3.72  (0.56) | 3.11  (0.53) | 3.52  (0.42) | 3.52  (0.38) | 3.80  (0.47) |
| **Attentional control** | 29.26  (5.39) | 31.39  (5.97) | 30.23  (5.69) | 32.33  (6.20) | 28.32  (6.30) | 31.10  (6.74) | 32.34  (5.44) | 33.14  (6.47) |
| **Trait mindfulness** | 39.42  (5.55) | 41.16  (5.93) | 41.23  (6.25) | 43.77  (6.49) | 38.81  (7.75) | 41.03  (5.84) | 41.79  (6.29) | 43.66  (6.81) |
| **Observing** | 9.45  (2.10) | 10.81  (2.71) | 10.33  (2.34) | 11.17  (2.21) | 9.39  (2.59) | 10.55  (2.17) | 10.66  (2.73) | 11.72  (2.07) |
| **Describing** | 10.35  (2.35) | 10.52  (2.58) | 11.03  (2.16) | 11.63  (2.31) | 10.68  (2.66) | 10.71  (2.30) | 11.31  (2.61) | 11.28  (2.17) |
| **Acting with awareness** | 9.13  (2.08) | 9.19  (2.30) | 9.07  (1.98) | 9.80  (2.07) | 8.77  (2.85) | 9.32  (2.64) | 9.38  (2.40) | 9.76  (2.37) |
| **Non-judgement** | 11.45  (2.38) | 11.29  (2.47) | 11.57  (2.62) | 12.10  (2.37) | 10.77  (3.33) | 11.58  (2.86) | 11.14  (2.63) | 12.17  (2.49) |
| **Non-reactivity** | 8.48  (2.32) | 10.16  (2.00) | 9.57  (2.14) | 10.23  (2.25) | 8.58  (2.72) | 9.42  (2.39) | 9.97  (1.86) | 10.45  (2.08) |
| **Noticing** | 2.72  (0.89) | 3.23  (0.78) | 3.35  (1.08) | 3.73  (0.75) | 2.90  (1.18) | 3.40  (0.91) | 3.16  (0.95) | 3.48  (0.74) |
| **Attentional regulation** | 2.31  (0.88) | 2.91  (1.04) | 2.74  (1.08) | 3.30  (0.87) | 2.20  (1.15) | 2.87  (1.00) | 2.90  (1.11) | 3.41  (0.84) |
| **Trusting** | 3.06  (1.14) | 3.56  (0.90) | 3.48  (1.20) | 4.04  (0.94) | 3.04  (1.12) | 3.49  (1.18) | 3.47  (1.10) | 3.82  (0.91) |
| **Body listening** | 2.20  (1.06) | 2.82  (1.29) | 2.42  (1.50) | 3.13  (1.30) | 1.94  (1.24) | 2.96  (1.02) | 2.46  (1.03) | 3.22  (1.19) |
| **Emotional awareness** | 2.92  (1.10) | 3.45  (1.26) | 3.57  (1.08) | 3.95  (0.81) | 3.04  (1.26) | 3.74  (1.00) | 3.06  (1.19) | 3.75  (0.87) |

This table depicts the pre-post means (standard deviations) for each experimental group pre-post two weeks of daily mindfulness practice [T1-T4] for Study 2 using a complete-case approach. *One case was missing from the anxiety measure and therefore 120 cases were included in total. This case was missing within the pre-scores for the befriending group and therefore 28 cases were included for the pre-score calculations instead of 29 for the anxiety measure.

1. **The strong association criterion – Study 2**

**Associations between change in candidate mechanisms and change in outcomes within the whole sample (Study 2)**

| **Mediator**  (Differential scores) | **Outcome**  (Differential scores) | **Time points** | **Output** |
| --- | --- | --- | --- |
| Trait self-compassion | Psychological quality of life | T1-T4 | r _(118)_ =0.40 [0.24, 0.54], p < 0.001 |
| Trait self-compassion | Anxiety | T1-T4 | r _(117)_ = -0.12 [-0.06, 0.29], p = 0.197 |
| Trait self-compassion | Depression | T1-T4 | r _(118)_ = -0.02 [-0.20, 0.16], p = 0.845 |
| Trait mindfulness | Psychological quality of life | T1-T4 | r _(118)_ = 0.38 [0.22, 0.52], p < 0.001 |
| Trait mindfulness | Anxiety | T1-T4 | r _(117)_ = -0.12 [-0.29, 0.06], p = 0.186 |
| Trait mindfulness | Depression | T1-T4 | r _(118)_ = -0.15 [-0.32, 0.03], p = 0.110 |
| Observing | Psychological quality of life | T1-T4 | r _(118)_ = 0.16 [-0.02, 0.33], p = 0.092 |
| Observing | Anxiety | T1-T4 | r _(117)_ = 0.04 [-0.14, 0.22], p = 0.686 |
| Observing | Depression | T1-T4 | r _(118)_ = 0.01 [-0.17, 0.19], p = 0.910 |
| Describing | Psychological quality of life | T1-T4 | r _(118)_ = 0.14 [-0.04, 0.31], p = 0.127 |
| Describing | Anxiety | T1-T4 | r _(117)_ = -0.02 [-0.2, 0.16], p = 0.796 |
| Describing | Depression | T1-T4 | r _(118)_ = 0.04 [-0.14, 0.22], p = 0.670 |
| Acting with awareness | Psychological quality of life | T1-T4 | r _(118)_ = 0.22 [0.04, 0.38], p = 0.015 |
| Acting with awareness | Anxiety | T1-T4 | r _(117)_ = -0.14 [-0.31, 0.04], p = 0.139 |
| Acting with awareness | Depression | T1-T4 | r _(118)_ = -0.30 [-0.46, -0.13], p < 0.001 |
| Non-judgment | Psychological quality of life | T1-T4 | r _(118)_ = 0.35 [0.18, 0.50], p < 0.001 |
| Non-judgement | Anxiety | T1-T4 | r _(117)_ = 0.01 [-0.17, 0.19], p = 0.939 |
| Non-judgement | Depression | T1-T4 | r _(118)_ = -0.06 [-0.24, 0.12], p = 0.512 |
| Non-reactivity | Psychological quality of life | T1-T4 | r _(118)_ = 0.16 [-0.02, 0.33], p = 0.085 |
| Non-reactivity | Anxiety | T1-T4 | r _(117)_ = -0.14 [-0.31, 0.04], p = 0.132 |
| Non-reactivity | Depression | T1-T4 | r _(118)_ = -0.01 [-0.19, 0.17], p = 0.911 |
| Trait decentering | Psychological quality of life | T1-T4 | r _(118)_ = 0.29 [0.20, 0.45], p = 0.001 |
| Trait decentering | Anxiety | T1-T4 | r _(117)_ = -0.06 [-0.24, 0.12], p = 0.540 |
| Trait decentering | Depression | T1-T4 | r _(118)_ = -0.06 [-0.24, 0.12], p = 0.494 |
| Attentional control | Psychological quality of life | T1-T4 | r _(118)_ = 0.10 [-0.08, 0.28], p = 0.259 |
| Attentional control | Anxiety | T1-T4 | r _(117)_ = -0.01 [-0.19, 0.17], p = 0.905 |
| Attentional control | Depression | T1-T4 | r _(118)_ = 0.01 [-0.17, 0.19], p = 0.912 |
| Noticing | Psychological quality of life | T1-T4 | r _(118)_ = 0.03 [-0.15, 0.21], p = 0.774 |
| Noticing | Anxiety | T1-T4 | r _(117)_ = -0.03 [-0.21, 0.15], p = 0.731 |
| Noticing | Depression | T1-T4 | r _(118)_ = 0.14 [-0.04, 0.31], p = 0.142 |
| Attentional regulation | Psychological quality of life | T1-T4 | r _(118)_ = 0.16 [-0.02, 0.33], p = 0.089 |
| Attentional regulation | Anxiety | T1-T4 | r _(117)_ = 0.05 [-0.13, 0.23], p = 0.579 |
| Attentional regulation | Depression | T1-T4 | r _(118)_ = 0.14 [-0.04, 0.31], p = 0.123 |
| Emotional awareness | Psychological quality of life | T1-T4 | r _(118)_ = 0.06 [-0.12, 0.24], p = 0.509 |
| Emotional awareness | Anxiety | T1-T4 | r _(117)_ = -0.04 [-0.22, 0.14], p = 0.669 |
| Emotional awareness | Depression | T1-T4 | r _(118)_ = -0.02 [-0.20, 0.16], p = 0.839 |
| Trusting | Psychological quality of life | T1-T4 | r _(118)_ = 0.16 [-0.02, 0.33], p = 0.073 |
| Trusting | Anxiety | T1-T4 | r _(117)_ = 0.01 [-0.17, 0.19], p = 0.921 |
| Trusting | Depression | T1-T4 | r _(118)_ = 0.05 [-0.13, 0.23], p = 0.626 |
| Body listening | Psychological quality of life | T1-T4 | r _(118)_ = 0.23 [0.05, 0.39], p = 0.014 |
| Body listening | Anxiety | T1-T4 | r _(117)_ = -0.07 [-0.25, 0.11], p = 0.453 |
| Body listening | Depression | T1-T4 | r _(118)_ = -0.01 [-0.19, 0.17], p = 0.889 |

This table depicts the non-parametric partial rank correlations within the whole sample (Study 2) for the associations between the differences in scores for candidate mechanisms and the differences in scores for proposed outcomes whilst controlling for baseline levels in outcomes. Partial rank correlations were calculated (Conover, 1999). Estimates for the confidence intervals for the r-values were calculated using this online calculator here: <http://vassarstats.net/rho.html>. The size of the associations (r-values) were interpreted using the conventions outlined by Hattie, J. (2011) [0.00-0.10 = no effect, 0.10-0.20= small effect, 0.20-0.30 = moderate effect, r > 0.30 = large effect] (Hattie, 2011).

**Associations between change in proposed mediators and change in outcomes within the body scan group (Study 2)**

| **Mediator**  (Differential scores) | **Outcome**  (Differential scores) | **Time points** | **Output** |
| --- | --- | --- | --- |
| Trait self-compassion | Psychological quality of life | T1-T4 | r _(28)_ = 0.39 [0.03, 0.66], p = 0.031 |
| Trait self-compassion | Anxiety | T1-T4 | r _(28)_ = 0.18 [-0.20, 0.51], p = 0.335 |
| Trait self-compassion | Depression | T1-T4 | r _(28)_ = 0.04 [-0.33, 0.40], p = 0.840 |
| Trait mindfulness | Psychological quality of life | T1-T4 | r _(28)_ = 0.42 [0.06, 0.68], p = 0.022 |
| Trait mindfulness | Anxiety | T1-T4 | r _(28)_ = 0.01 [-0.36, 0.38], p = 0.946 |
| Trait mindfulness | Depression | T1-T4 | r _(28)_ = -0.37 [-0.65, -0.01], p = .046 |
| Observing | Psychological quality of life | T1-T4 | r _(28)_ = 0.38 [0.02, 0.66], p = 0.038 |
| Observing | Anxiety | T1-T4 | r _(28)_ = 0.05 [-0.32, 0.41], p = 0.812 |
| Observing | Depression | T1-T4 | r _(28)_ = -0.14 [-0.48, 0.24], p = 0.453 |
| Describing | Psychological quality of life | T1-T4 | r _(28)_ = 0.20 [-0.18, 0.53], p = 0.287 |
| Describing | Anxiety | T1-T4 | r _(28)_ = 0.13 [-0.25, 0.47], p = 0.492 |
| Describing | Depression | T1-T4 | r _(28)_ = -0.22 [-0.54, 0.16], p = 0.246 |
| Acting with awareness | Psychological quality of life | T1-T4 | r _(28)_ = 0.34 [-0.03, 0.63], p = 0.070 |
| Acting with awareness | Anxiety | T1-T4 | r _(28)_ = -0.12 [-0.47, 0.26], p = 0.545 |
| Acting with awareness | Depression | T1-T4 | r _(28)_ = -0.60 [-0.79, -0.30], p < 0.001 |
| Non-judgment | Psychological quality of life | T1-T4 | r _(28)_ = 0.59 [0.29, 0.79], p < 0.001 |
| Non-judgement | Anxiety | T1-T4 | r _(28)_ = 0.10 [-0.28, 0.45], p = 0.598 |
| Non-judgement | Depression | T1-T4 | r _(28)_ = -0.34 [-0.63, 0.03], p = 0.065 |
| Non-reactivity | Psychological quality of life | T1-T4 | r _(28)_ = 0.00 [-0.37, 0.37], p = 0.990 |
| Non-reactivity | Anxiety | T1-T4 | r _(28)_ = -0.11 [-0.46, 0.27], p = 0.554 |
| Non-reactivity | Depression | T1-T4 | r _(28)_ = 0.16 [-0.22, 0.50], p = 0.415 |
| Trait decentering | Psychological quality of life | T1-T4 | r _(28)_ = 0.44 [0.09, 0.69], p = 0.014 |
| Trait decentering | Anxiety | T1-T4 | r _(28)_ = 0.07 [-0.30, 0.43], p = 0.706 |
| Trait decentering | Depression | T1-T4 | r _(28)_ = -0.08 [-0.43, 0.30], p = 0.667 |
| Attentional control | Psychological quality of life | T1-T4 | r _(28)_ = 0.24 [-0.14, 0.56], p = 0.200 |
| Attentional control | Anxiety | T1-T4 | r _(28)_ = 0.18 [-0.20, 0.51], p = 0.345 |
| Attentional control | Depression | T1-T4 | r _(28)_ = 0.11 [-0.27, 0.46], p = 0.549 |
| Noticing | Psychological quality of life | T1-T4 | r _(28)_ = 0.14 [-0.24, 0.48], p = 0.472 |
| Noticing | Anxiety | T1-T4 | r _(28)_ = -0.18 [-0.51, 0.20], p = 0.332 |
| Noticing | Depression | T1-T4 | r _(28)_ = 0.12 [-0.26, 0.47], p = 0.527 |
| Attentional regulation | Psychological quality of life | T1-T4 | r _(28)_ = 0.09 [-0.29, 0.44], p = 0.642 |
| Attentional regulation | Anxiety | T1-T4 | r _(28)_ = 0.16 [-0.22, 0.50], p = 0.410 |
| Attentional regulation | Depression | T1-T4 | r _(28)_ = 0.05 [-0.32, 0.41], p = 0.807 |
| Emotional awareness | Psychological quality of life | T1-T4 | r _(28)_ = 0.27 [-0.11, 0.58], p = 0.152 |
| Emotional awareness | Anxiety | T1-T4 | r _(28)_ = 0.04 [-0.33, 0.4], p = 0.835 |
| Emotional awareness | Depression | T1-T4 | r _(28)_ = -0.04 [-.04, 0.33], p = 0.849 |
| Trusting | Psychological quality of life | T1-T4 | r _(28)_ = 0.12 [-0.26, 0.47], p = 0.523 |
| Trusting | Anxiety | T1-T4 | r _(28)_ = 0.12 [-0.26, 0.47], p = 0.546 |
| Trusting | Depression | T1-T4 | r _(28)_ = 0.04 [-0.33, 0.40], p = 0.821 |
| Body listening | Psychological quality of life | T1-T4 | r _(28)_ = 0.23 [-0.15, 0.55], p = 0.220 |
| Body listening | Anxiety | T1-T4 | r _(28)_ = 0.04 [-0.33, 0.4], p = 0.830 |
| Body listening | Depression | T1-T4 | r _(28)_ = -0.07 [-0.43, 0.30], p = 0.728 |

This table depicts the non-parametric partial rank correlations within the body scan group only (Study 2) for the associations between the differences in scores for candidate mechanisms and the differences in scores for proposed outcomes whilst controlling for baseline levels in outcomes. Partial rank correlations were calculated (Conover, 1999). Estimates for the confidence intervals for the r-values were calculated using this online calculator here: <http://vassarstats.net/rho.html>. The size of the associations (r-values) were interpreted using the conventions outlined by Hattie, J. (2011) [0.00-0.10 = no effect, 0.10-0.20= small effect, 0.20-0.30 = moderate effect, r > 0.30 = large effect] (Hattie, 2011).

**Associations between change in proposed mediators and change in outcomes within the mindful movement group (Study 2)**

| **Mediator**  (Differential scores) | **Outcome**  (Differential scores) | **Time points** | **Output** |
| --- | --- | --- | --- |
| Trait self-compassion | Psychological quality of life | T1-T4 | r _(27)_ = 0.31 [-0.07, 0.61], p = 0.104 |
| Trait self-compassion | Anxiety | T1-T4 | r _(27)_ = -0.28 [-0.59, 0.10], p = 0.149 |
| Trait self-compassion | Depression | T1-T4 | r _(27)_ = -0.02 [-0.39, 0.36], p = 0.921 |
| Trait mindfulness | Psychological quality of life | T1-T4 | r _(27)_ = 0.34 [-0.04, 0.63], p = 0.073 |
| Trait mindfulness | Anxiety | T1-T4 | r _(27)_ = -0.38 [-0.66,-0.01], p = 0.045 |
| Trait mindfulness | Depression | T1-T4 | r _(27)_ = -0.12 [-0.47, 0.26], p = 0.545 |
| Observing | Psychological quality of life | T1-T4 | r _(27)_ = -0.08 [-0.44, 0.30], p = 0.668 |
| Observing | Anxiety | T1-T4 | r _(27)_ = 0.01 [-0.36, 0.38], p = 0.950 |
| Observing | Depression | T1-T4 | r _(27)_ = -0.01 [-0.38, 0.36], p = 0.977 |
| Describing | Psychological quality of life | T1-T4 | r _(27)_ = 0.11 [-0.27, 0.46], p = 0.570 |
| Describing | Anxiety | T1-T4 | r _(27)_ = 0.05 [-0.33, 0.42], p = 0.818 |
| Describing | Depression | T1-T4 | r _(27)_ = 0.09 [-0.29, 0.45], p = 0.642 |
| Acting with awareness | Psychological quality of life | T1-T4 | r _(27)_ = 0.28 [-0.10, 0.59], p = 0.141 |
| Acting with awareness | Anxiety | T1-T4 | r _(27)_ = -0.49 [-0.73, -0.14], p = 0.007 |
| Acting with awareness | Depression | T1-T4 | r _(27)_ = -0.26 [-0.58, 0.13], p = 0.170 |
| Non-judgment | Psychological quality of life | T1-T4 | r _(27)_ = 0.31 [-0.07, 0.61], p = 0.108 |
| Non-judgement | Anxiety | T1-T4 | r _(27)_ = -0.16 [-0.50, 0.23], p = 0.409 |
| Non-judgement | Depression | T1-T4 | r _(27)_ = -0.06 [-0.42, 0.32], p = 0.751 |
| Non-reactivity | Psychological quality of life | T1-T4 | r _(27)_ = 0.18 [-0.21, 0.52], p = 0.359 |
| Non-reactivity | Anxiety | T1-T4 | r _(27)_ = -0.22 [-0.55, 0.17], p = 0.246 |
| Non-reactivity | Depression | T1-T4 | r _(27)_ = -0.00 [-0.37, 0.37], p = 0.994 |
| Trait decentering | Psychological quality of life | T1-T4 | r _(27)_ = 0.13 [-0.26, 0.48], p = 0.494 |
| Trait decentering | Anxiety | T1-T4 | r _(27)_ = -0.17 [-0.51, 0.22], p = 0.390 |
| Trait decentering | Depression | T1-T4 | r _(27)_ = -0.13 [-0.48, 0.26], p = 0.499 |
| Attentional control | Psychological quality of life | T1-T4 | r _(27)_ = 0.14 [-0.25, 0.49], p = 0.484 |
| Attentional control | Anxiety | T1-T4 | r _(27)_ = -0.17 [-0.51, 0.22], p = 0.392 |
| Attentional control | Depression | T1-T4 | r _(27)_ = -0.05 [-0.42, 0.33], p = 0.795 |
| Noticing | Psychological quality of life | T1-T4 | r _(27)_ = -0.00 [-0.37, 0.37], p = 0.991 |
| Noticing | Anxiety | T1-T4 | r _(27)_ = -0.10 [-0.46, 0.28], p = 0.592 |
| Noticing | Depression | T1-T4 | r _(27)_ = 0.11 [-0.27, 0.46], p = 0.583 |
| Attentional regulation | Psychological quality of life | T1-T4 | r _(27)_ = 0.15 [-0.24, 0.50], p = 0.425 |
| Attentional regulation | Anxiety | T1-T4 | r _(27)_ = -0.01 [-0.38, 0.36], p = 0.950 |
| Attentional regulation | Depression | T1-T4 | r _(27)_ = -0.11 [-0.46, 0.27], p = 0.570 |
| Emotional awareness | Psychological quality of life | T1-T4 | r _(27)_ = -0.07 [-0.43, 0.31], p = 0.733 |
| Emotional awareness | Anxiety | T1-T4 | r _(27)_ = -0.26 [-0.58, 0.13], p = 0.174 |
| Emotional awareness | Depression | T1-T4 | r _(27)_ = 0.08 [-0.30, 0.44], p = 0.666 |
| Trusting | Psychological quality of life | T1-T4 | r _(27)_ = -0.00 [-0.37, 0.37], p = 0.988 |
| Trusting | Anxiety | T1-T4 | r _(27)_ = 0.12 [-0.26, 0.47], p = 0.549 |
| Trusting | Depression | T1-T4 | r _(27)_ = 0.21 [-0.8, 0.54], p = 0.285 |
| Body listening | Psychological quality of life | T1-T4 | r _(27)_ = 0.20 [-0.19, 0.53], p = 0.307 |
| Body listening | Anxiety | T1-T4 | r _(27)_ = -0.16 [-0.50, 0.23], p = 0.415 |
| Body listening | Depression | T1-T4 | r _(27)_ = -0.20 [-0.53, 0.19], p = 0.293 |

This table depicts the non-parametric partial rank correlations within the mindful movement group only (Study 2) for the associations between the differences in scores for candidate mechanisms and the differences in scores for proposed outcomes whilst controlling for baseline levels in outcomes. Partial rank correlations were calculated (Conover, 1999). Estimates for the confidence intervals for the r-values were calculated using this online calculator here: <http://vassarstats.net/rho.html>. The size of the associations (r-values) were interpreted using the conventions outlined by Hattie, J. (2011) [0.00-0.10 = no effect, 0.10-0.20= small effect, 0.20-0.30 = moderate effect, r > 0.30 = large effect] (Hattie, 2011).

**Associations between change in proposed mediators and change in outcomes within the breath and body group (Study 2)**

| **Mediator**  (Differential scores) | **Outcome**  (Differential scores) | **Time points** | **Output** |
| --- | --- | --- | --- |
| Trait self-compassion | Psychological quality of life | T1-T4 | r _(28)_ = 0.33 [-0.04, 0.62], p = 0.080 |
| Trait self-compassion | Anxiety | T1-T4 | r _(28)_ = -0.25 [-0.56, 0.13], p = 0.180 |
| Trait self-compassion | Depression | T1-T4 | r _(28)_ = -0.01 [-0.38, 0.36], p = 0.952 |
| Trait mindfulness | Psychological quality of life | T1-T4 | r _(28)_ = 0.35 [-0.02, 0.64], p = 0.055 |
| Trait mindfulness | Anxiety | T1-T4 | r _(28)_ = -0.01 [-0.38, 0.36], p = 0.949 |
| Trait mindfulness | Depression | T1-T4 | r _(28)_ = 0.10 [-0.28, 0.45], p = 0.590 |
| Observing | Psychological quality of life | T1-T4 | r _(28)_ = 0.36 [-0.01, 0.64], p = 0.049 |
| Observing | Anxiety | T1-T4 | r _(28)_ = 0.06 [-0.31, 0.42], p = 0.763 |
| Observing | Depression | T1-T4 | r _(28)_ = 0.05 [-0.32, 0.41], p = 0.783 |
| Describing | Psychological quality of life | T1-T4 | r _(28)_ = 0.16 [-0.22, 0.50], p = 0.404 |
| Describing | Anxiety | T1-T4 | r _(28)_ = 0.05 [-0.32, 0.41], p = 0.806 |
| Describing | Depression | T1-T4 | r _(28)_ = 0.29 [-0.09, 0.59], p = 0.119 |
| Acting with awareness | Psychological quality of life | T1-T4 | r _(28)_ = 0.31 [-0.06, 0.61], p = 0.099 |
| Acting with awareness | Anxiety | T1-T4 | r _(28)_ = -0.03 [-0.39, 0.34], p = 0.870 |
| Acting with awareness | Depression | T1-T4 | r _(28)_ = -0.20 [-0.53, 0.18], p = 0.291 |
| Non-judgment | Psychological quality of life | T1-T4 | r _(28)_ = 0.07 [-0.30, 0.43], p = 0.732 |
| Non-judgement | Anxiety | T1-T4 | r _(28)_ = -0.10 [-0.45, 0.28], p = 0.614 |
| Non-judgement | Depression | T1-T4 | r _(28)_ = 0.02 [-0.35, 0.38], p = 0.905 |
| Non-reactivity | Psychological quality of life | T1-T4 | r _(28)_ = 0.31 [-0.06, 0.61], p = 0.101 |
| Non-reactivity | Anxiety | T1-T4 | r _(28)_ = 0.02 [-0.35, 0.38], p = 0.930 |
| Non-reactivity | Depression | T1-T4 | r _(28)_ = 0.20 [-0.18, 0.53], p = 0.303 |
| Trait decentering | Psychological quality of life | T1-T4 | r _(28)_ = 0.31 [-0.06, 0.61], p = 0.098 |
| Trait decentering | Anxiety | T1-T4 | r _(28)_ = -0.02 [-0.38, 0.35], p = 0.900 |
| Trait decentering | Depression | T1-T4 | r _(28)_ = -0.05 [-0.41, 0.32], p = 0.779 |
| Attentional control | Psychological quality of life | T1-T4 | r _(28)_ = 0.13 [-0.25, 0.47], p = 0.494 |
| Attentional control | Anxiety | T1-T4 | r _(28)_ = -0.07 [-0.43, 0.30], p = 0.732 |
| Attentional control | Depression | T1-T4 | r _(28)_ = -0.08 [-0.43, 0.30], p = 0.668 |
| Noticing | Psychological quality of life | T1-T4 | r _(28)_ = 0.18 [-0.20, 0.51], p = 0.337 |
| Noticing | Anxiety | T1-T4 | r _(28)_ = 0.16 [-0.22, 0.50], p = 0.412 |
| Noticing | Depression | T1-T4 | r _(28)_ = 0.26 [-0.12, 0.57], p = 0.171 |
| Attentional regulation | Psychological quality of life | T1-T4 | r _(28)_ = 0.27 [-0.11, 0.58], p = 0.142 |
| Attentional regulation | Anxiety | T1-T4 | r _(28)_ = 0.08 [-0.30, 0.43], p = 0.692 |
| Attentional regulation | Depression | T1-T4 | r _(28)_ = 0.24 [-0.14, 0.56], p = 0.200 |
| Emotional awareness | Psychological quality of life | T1-T4 | r _(28)_ = 0.10 [-0.28, 0.45], p = 0.584 |
| Emotional awareness | Anxiety | T1-T4 | r _(28)_ = 0.01 [-0.36, 0.38], p = 0.967 |
| Emotional awareness | Depression | T1-T4 | r _(28)_ = -0.06 [-0.42, 0.31], p = 0.761 |
| Trusting | Psychological quality of life | T1-T4 | r _(28)_ = 0.29 [-0.09, 0.59], p = 0.124 |
| Trusting | Anxiety | T1-T4 | r _(28)_ = 0.09 [-0.29, 0.44], p = 0.642 |
| Trusting | Depression | T1-T4 | r _(28)_ = 0.11 [-0.27, 0.46], p = 0.567 |
| Body listening | Psychological quality of life | T1-T4 | r _(28)_ = 0.05 [-0.32, 0.41], p = 0.798 |
| Body listening | Anxiety | T1-T4 | r _(28)_ = -0.06 [-0.42, 0.31], p = 0.771 |
| Body listening | Depression | T1-T4 | r _(28)_ = 0.23 [-0.15, 0.55], p = 0.213 |

This table depicts the non-parametric partial rank correlations within the breath and body group only (Study 2) for the associations between the differences in scores for candidate mechanisms and the differences in scores for proposed outcomes whilst controlling for baseline levels in outcomes. Partial rank correlations were calculated (Conover, 1999). Estimates for the confidence intervals for the r-values were calculated using this online calculator here: <http://vassarstats.net/rho.html>. The size of the associations (r-values) were interpreted using the conventions outlined by Hattie, J. (2011) [0.00-0.10 = no effect, 0.10-0.20= small effect, 0.20-0.30 = moderate effect, r > 0.30 = large effect] (Hattie, 2011).

**Associations between change in proposed mediators and change in outcomes within the befriending group (Study 2)**

| **Mediator**  (Differential scores) | **Outcome**  (Differential scores) | **Time points** | **Output** |
| --- | --- | --- | --- |
| Trait self-compassion | Psychological quality of life | T1-T4 | r _(26)_ = 0.50 [0.15, 0.74], p = 0.007 |
| Trait self-compassion | Anxiety | T1-T4 | r _(25)_ = -0.06 [-0.44, 0.34], p = 0.757 |
| Trait self-compassion | Depression | T1-T4 | r _(26)_ = -0.02 [-0.40, 0.36], p = 0.914 |
| Trait mindfulness | Psychological quality of life | T1-T4 | r _(26)_ = 0.41 [0.04, 0.68], p = 0.032 |
| Trait mindfulness | Anxiety | T1-T4 | r _(25)_ = -0.10 [-0.47, 0.30], p = 0.604 |
| Trait mindfulness | Depression | T1-T4 | r _(26)_ = -0.12 [-0.48, 0.27], p = 0.539 |
| Observing | Psychological quality of life | T1-T4 | r _(26)_ = -0.01 [-0.39, 0.37], p = 0.982 |
| Observing | Anxiety | T1-T4 | r _(25)_ = -0.17 [-0.52, 0.23], p = 0.388 |
| Observing | Depression | T1-T4 | r _(26)_ = 0.12 [-0.27, 0.48], p = 0.553 |
| Describing | Psychological quality of life | T1-T4 | r _(26)_ = -0.09 [-0.45, 0.30], p = 0.644 |
| Describing | Anxiety | T1-T4 | r _(25)_ = -0.06 [-0.44, 0.34], p = 0.755 |
| Describing | Depression | T1-T4 | r _(26)_ = 0.15 [-0.24, 0.50], p = 0.438 |
| Acting with awareness | Psychological quality of life | T1-T4 | r _(26)_ = 0.02 [-0.36, 0.40], p = 0.915 |
| Acting with awareness | Anxiety | T1-T4 | r _(25)_ = 0.03 [-0.36, 0.41], p = 0.902 |
| Acting with awareness | Depression | T1-T4 | r _(26)_ = -0.21 [-0.55, 0.18], p = 0.292 |
| Non-judgment | Psychological quality of life | T1-T4 | r _(26)_ = 0.41 [0.04, 0.68], p = 0.031 |
| Non-judgement | Anxiety | T1-T4 | r _(25)_ = 0.16 [-0.24, 0.52], p = 0.430 |
| Non-judgement | Depression | T1-T4 | r _(26)_ = 0.18 [-0.21, 0.52], p = 0.366 |
| Non-reactivity | Psychological quality of life | T1-T4 | r _(26)_ = 0.23 [-0.16, 0.56], p = 0.248 |
| Non-reactivity | Anxiety | T1-T4 | r _(25)_ = -0.30 [-0.61, 0.09], p = 0.129 |
| Non-reactivity | Depression | T1-T4 | r _(26)_ = -0.45 [-0.71, -0.09], p = 0.017 |
| Trait decentering | Psychological quality of life | T1-T4 | r _(26)_ = 0.21 [-0.18, 0.55], p = 0.274 |
| Trait decentering | Anxiety | T1-T4 | r _(25)_ = 0.04 [-0.35, 0.42], p = 0.848 |
| Trait decentering | Depression | T1-T4 | r _(26)_ = 0.07 [-0.32, 0.44], p = 0.723 |
| Attentional control | Psychological quality of life | T1-T4 | r _(26)_ = -0.05 [-0.42, 0.34], p = 0.817 |
| Attentional control | Anxiety | T1-T4 | r _(25)_ = -0.01 [-0.40, 0.38], p = 0.974 |
| Attentional control | Depression | T1-T4 | r _(26)_ = 0.16 [-0.23, 0.51], p = 0.432 |
| Noticing | Psychological quality of life | T1-T4 | r _(26)_ = -0.21 [-0.55, 0.18], p = 0.280 |
| Noticing | Anxiety | T1-T4 | r _(25)_ = -0.12 [-0.48, 0.28], p = 0.555 |
| Noticing | Depression | T1-T4 | r _(26)_ = -0.13 [-0.49, 0.26], p = 0.521 |
| Attentional regulation | Psychological quality of life | T1-T4 | r _(26)_ = 0.06 [-0.33, 0.43], p = 0.759 |
| Attentional regulation | Anxiety | T1-T4 | r _(25)_ = 0.07 [-0.33, 0.45], p = 0.747 |
| Attentional regulation | Depression | T1-T4 | r _(26)_ = 0.32 [-0.07, 0.62], p = 0.095 |
| Emotional awareness | Psychological quality of life | T1-T4 | r _(26)_ = -0.03 [-0.41, 0.35], p = 0.867 |
| Emotional awareness | Anxiety | T1-T4 | r _(25)_ = -0.01 [-0.40, 0.38], p = 0.971 |
| Emotional awareness | Depression | T1-T4 | r _(26)_ = -0.23 [-0.56, 0.16], p = 0.234 |
| Trusting | Psychological quality of life | T1-T4 | r _(26)_ = 0.18 [-0.21, 0.52], p = 0.356 |
| Trusting | Anxiety | T1-T4 | r _(25)_ = -0.24 [-0.57, 0.16], p = 0.232 |
| Trusting | Depression | T1-T4 | r _(26)_ = -0.15 [-0.50, 0.24], p = 0.444 |
| Body listening | Psychological quality of life | T1-T4 | r _(26)_ = 0.35 [-0.03, 0.64], p = 0.066 |
| Body listening | Anxiety | T1-T4 | r _(25)_ = -0.08 [-0.45, 0.32], p = 0.699 |
| Body listening | Depression | T1-T4 | r _(26)_ = -0.26 [-0.58, 0.13], p = 0.181 |

This table depicts the non-parametric partial rank correlations within the befriending group only (Study 2) for the associations between the differences in scores for candidate mechanisms and the differences in scores for proposed outcomes whilst controlling for baseline levels in outcomes. Partial rank correlations were calculated (Conover, 1999). Estimates for the confidence intervals for the r-values were calculated using this online calculator here: <http://vassarstats.net/rho.html>. The size of the associations (r-values) were interpreted using the conventions outlined by Hattie, J. (2011) [0.00-0.10 = no effect, 0.10-0.20= small effect, 0.20-0.30 = moderate effect, r > 0.30 = large effect] (Hattie, 2011).

1. **The gradient criterion – Study 2**

**Associations between amount of self-reported practice and post-intervention scores for outcomes and proposed mechanisms within the whole sample (Study 2)**

| **Variables** | **Time points** | **Output** |
| --- | --- | --- |
| Self-compassion | T4 | r _(118)_ = 0.18 [0.00, 0.35], p = 0.053 |
| Mindfulness | T4 | r _(118)_ = 0.18 [0.00, 0.35], p = 0.056 |
| Observing | T4 | r _(118)_ = 0.15 [-0.03, 0.32], p = 0.095 |
| Describing | T4 | r _(118)_ = 0.09 [-0.09, 0.27], p = 0.355 |
| Acting with awareness | T4 | r _(118)_ = -0.02 [-0.20, 0.16], p = 0.830 |
| Non-judgment | T4 | r _(118)_ = 0.16 [-0.02, 0.33], p = 0.085 |
| Non-reactivity | T4 | r _(118)_ = 0.17 [-0.01, 0.34], p = 0.058 |
| Noticing | T4 | r _(118)_ = 0.22 [0.04, 0.38], p = 0.016 |
| Attentional regulation | T4 | r _(118)_ = 0.28 [0.11, 0.44], p = 0.002 |
| Trusting | T4 | r _(118)_ = 0.22 [0.04, 0.38], p = 0.015 |
| Body listening | T4 | r _(118)_ = 0.22 [0.04, 0.38], p = 0.014 |
| Emotional awareness | T4 | r _(118)_ = 0.22 [0.04, 0.38], p = 0.017 |
| Decentering | T4 | r _(118)_ = 0.23 [0.05, 0.39], p = 0.012 |
| Attentional control | T4 | r _(118)_ = 0.26 [0.08, 0.42], p = 0.005 |
| Psychological quality of life | T4 | r _(118)_ = 0.25 [0.07, 0.41], p = 0.007 |
| Depression | T4 | r _(118)_ = -0.06 [-0.24, 0.12], p = 0.528 |
| Anxiety | T4 | r _(117)_ = -0.12 [-0.29, 0.06], p = 0.196 |

This table depicts the partial rank correlations between the amount of practice and post-intervention scores for proposed mechanisms and outcomes, whilst controlling for baseline levels in the proposed mechanism or outcome within the whole sample (Study 2). Partial rank correlations were calculated (Conover, 1999). Estimates for the confidence intervals for the r-values were calculated using this online calculator here: <http://vassarstats.net/rho.html>. The size of the associations (r-values) were interpreted using the conventions outlined by Hattie, J. (2011) [0.00-0.10 = no effect, 0.10-0.20= small effect, 0.20-0.30 = moderate effect, r > 0.30 = large effect] (Hattie, 2011).

**Associations between amount of self-reported practice and post-intervention scores for outcomes and proposed mechanisms within the body scan group (Study 2)**

| **Variables** | **Time points** | **Output** |
| --- | --- | --- |
| Self-compassion | T4 | r _(28)_ = 0.12 [-0.26, 0.47], p = 0.513 |
| Mindfulness | T4 | r _(28)_ = 0.01 [-0.26, 0.38], p = 0.966 |
| Observing | T4 | r _(28)_ = 0.24 [-0.14, 0.56], p = 0.203 |
| Describing | T4 | r _(28)_ = 0.07 [-0.30, 0.43], p = 0.732 |
| Acting with awareness | T4 | r _(28)_ = -0.12 [-0.47, 0.26], p = 0.533 |
| Non-judgment | T4 | r _(28)_ = 0.00 [-0.37, 0.37], p = 0.993 |
| Non-reactivity | T4 | r _(28)_ = 0.07 [-0.30, 0.43], p = 0.734 |
| Noticing | T4 | r _(28)_ = 0.29 [-0.09, 0.59], p = 0.120 |
| Attentional regulation | T4 | r _(28)_ = 0.14 [-0.24, 0.48], p = 0.460 |
| Trusting | T4 | r _(28)_ = 0.12 [-0.26, 0.47], p = 0.519 |
| Body listening | T4 | r _(28)_ = 0.18 [-0.20, 0.51], p = 0.352 |
| Emotional awareness | T4 | r _(28)_ = 0.00 [-0.37, 0.37], p = 0.993 |
| Decentering | T4 | r _(28)_ = 0.21 [-0.17, 0.54], p = 0.270 |
| Attentional control | T4 | r _(28)_ = 0.26 [-0.12, 0.57], p = 0.166 |
| Psychological quality of life | T4 | r _(28)_ = 0.06 [-0.31, 0.42], p = 0.761 |
| Depression | T4 | r _(28)_ = -0.04 [-0.40, 0.33], p = 0.822 |
| Anxiety | T4 | r _(28)_ = 0.09 [-0.29, 0.44], p = 0.636 |

This table depicts the partial rank correlations between the amount of practice and post-intervention scores for proposed mechanisms and outcomes, whilst controlling for baseline levels in the proposed mechanism or outcome within the body scan group only (Study 2). Partial rank correlations were calculated (Conover, 1999). Estimates for the confidence intervals for the r-values were calculated using this online calculator here: <http://vassarstats.net/rho.html>. The size of the associations (r-values) were interpreted using the conventions outlined by Hattie, J. (2011) [0.00-0.10 = no effect, 0.10-0.20= small effect, 0.20-0.30 = moderate effect, r > 0.30 = large effect] (Hattie, 2011).

**Associations between amount of self-reported practice and post-intervention scores for outcomes and proposed mechanisms within the mindful movement group (Study 2)**

| **Variables** | **Time points** | **Output** |
| --- | --- | --- |
| Self-compassion | T4 | r _(27)_ = 0.32 [-0.06, 0.62], p = 0.087 |
| Mindfulness | T4 | r _(27)_ = 0.32 [-0.06, 0.62], p = 0.090 |
| Observing | T4 | r _(27)_ = 0.06 [-0.32, 0.42], p = 0.769 |
| Describing | T4 | r _(27)_ = 0.24 [-0.15, 0.56], p = 0.207 |
| Acting with awareness | T4 | r _(27)_ = 0.21 [-0.18, 0.54], p = 0.281 |
| Non-judgment | T4 | r _(27)_ = 0.12 [-0.26, 0.47], p = 0.547 |
| Non-reactivity | T4 | r _(27)_ = 0.36 [-0.02, 0.65], p = 0.052 |
| Noticing | T4 | r _(27)_ = 0.41 [0.04, 0.68], p = 0.026 |
| Attentional regulation | T4 | r _(27)_ = 0.38 [0.01, 0.66], p = 0.043 |
| Trusting | T4 | r _(27)_ = 0.25 [-0.14, 0.57], p = 0.187 |
| Body listening | T4 | r _(27)_ = 0.16 [-0.23, 0.50], p = 0.418 |
| Emotional awareness | T4 | r _(27)_ = 0.29 [-0.09, 0.60], p = 0.123 |
| Decentering | T4 | r _(27)_ = 0.40 [0.03, 0.67], p = 0.034 |
| Attentional control | T4 | r _(27)_ = 0.42 [0.06, 0.69], p = 0.025 |
| Psychological quality of life | T4 | r _(27)_ = 0.39 [0.02, 0.67], p = 0.036 |
| Depression | T4 | r _(27)_ = -0.25 [-0.57,0.14], p = 0.189 |
| Anxiety | T4 | r _(27)_ = -0.47 [-0.72, -0.12], p = 0.009 |

This table depicts the partial rank correlations between the amount of practice and post-intervention scores for proposed mechanisms and outcomes, whilst controlling for baseline levels in the proposed mechanism or outcome within the mindful movement group only (Study 2). Partial rank correlations were calculated (Conover, 1999). Estimates for the confidence intervals for the r-values were calculated using this online calculator here: <http://vassarstats.net/rho.html>. The size of the associations (r-values) were interpreted using the conventions outlined by Hattie, J. (2011) [0.00-0.10 = no effect, 0.10-0.20= small effect, 0.20-0.30 = moderate effect, r > 0.30 = large effect] (Hattie, 2011).

**Associations between amount of self-reported practice and post-intervention scores for outcomes and proposed mechanisms within the breath and body group (Study 2)**

| **Variables** | **Time points** | **Output** |
| --- | --- | --- |
| Self-compassion | T4 | r _(28)_ = 0.08 [-0.30, 0.43], p = 0.676 |
| Mindfulness | T4 | r _(28)_ = 0.18 [-0.20, 0.51], p = 0.343 |
| Observing | T4 | r _(28)_ = 0.35 [-0.02, 0.64], p = 0.055 |
| Describing | T4 | r _(28)_ = 0.15 [-0.23, 0.49], p = 0.428 |
| Acting with awareness | T4 | r _(28)_ = -0.02 [-0.38, 0.35], p = 0.902 |
| Non-judgment | T4 | r _(28)_ = 0.18 [-0.20, 0.51], p = 0.346 |
| Non-reactivity | T4 | r _(28)_ = 0.31 [-0.06, 0.61], p = 0.098 |
| Noticing | T4 | r _(28)_ = 0.21 [-0.17, 0.54], p = 0.269 |
| Attentional regulation | T4 | r _(28)_ = 0.45 [0.10, 0.70], p = 0.014 |
| Trusting | T4 | r _(28)_ = 0.36 [-0.01, 0.64], p = 0.054 |
| Body listening | T4 | r _(28)_ = 0.33 [-0.04, 0.62], p = 0.080 |
| Emotional awareness | T4 | r _(28)_ = 0.45 [0.10, 0.70], p = 0.013 |
| Decentering | T4 | r _(28)_ = 0.12 [-0.26, 0.47], p = 0.545 |
| Attentional control | T4 | r _(28)_ = 0.17 [-0.21, 0.51], p = 0.384 |
| Psychological quality of life | T4 | r _(28)_ = 0.14 [-0.24, 0.48], p = 0.462 |
| Depression | T4 | r _(28)_ = -0.11 [-0.46, 0.27], p = 0.573 |
| Anxiety | T4 | r _(28)_ = 0.06 [-0.31, 0.42], p = 0.735 |

This table depicts the partial rank correlations between the amount of practice and post-intervention scores for proposed mechanisms and outcomes, whilst controlling for baseline levels in the proposed mechanism or outcome within the breath and body group only (Study 2). Partial rank correlations were calculated (Conover, 1999). Estimates for the confidence intervals for the r-values were calculated using this online calculator here: <http://vassarstats.net/rho.html>. The size of the associations (r-values) were interpreted using the conventions outlined by Hattie, J. (2011) [0.00-0.10 = no effect, 0.10-0.20= small effect, 0.20-0.30 = moderate effect, r > 0.30 = large effect] (Hattie, 2011).

**Associations between amount of self-reported practice and post-intervention scores for outcomes and proposed mechanisms within the befriending group (Study 2)**

| **Variables** | **Time points** | **Output** |
| --- | --- | --- |
| Self-compassion | T4 | r _(26)_ = 0.25 [-0.14, 0.58], p = 0.192 |
| Mindfulness | T4 | r _(26)_ = -0.02 [-0.40, 0.36], p = 0.907 |
| Observing | T4 | r _(26)_ = -0.08 [-0.45, 0.31], p = 0.695 |
| Describing | T4 | r _(26)_ = -0.07 [-0.44, 0.32], p = 0.714 |
| Acting with awareness | T4 | r _(26)_ = -0.37 [-0.66, 0.01], p = 0.053 |
| Non-judgment | T4 | r _(26)_ = 0.18 [-0.21, 0.52], p = 0.370 |
| Non-reactivity | T4 | r _(26)_ = -0.17 [-0.52, 0.22], p = 0.385 |
| Noticing | T4 | r _(26)_ = -0.10 [-0.46, 0.29], p = 0.622 |
| Attentional regulation | T4 | r _(26)_ = 0.17 [-0.22, 0.52], p = 0.381 |
| Trusting | T4 | r _(26)_ = 0.17 [-0.22, 0.52], p = 0.403 |
| Body listening | T4 | r _(26)_ = 0.27 [-0.12, 0.59], p = 0.172 |
| Emotional awareness | T4 | r _(26)_ = 0.05 [-0.34, 0.42], p = 0.801 |
| Decentering | T4 | r _(26)_ = -0.02 [-0.40, 0.36], p = 0.941 |
| Attentional control | T4 | r _(26)_ = 0.10 [-0.29, 0.46], p = 0.620 |
| Psychological quality of life | T4 | r _(26)_ = 0.53 [0.19, 0.76], p = 0.004 |
| Depression | T4 | r _(26)_ = -0.08 [-0.45, 0.31], p = 0.700 |
| Anxiety | T4 | r _(25)_ = -0.24 [-0.16, 0.57], p = 0.227 |

This table depicts the partial rank correlations between the amount of practice and post-intervention scores for proposed mechanisms and outcomes, whilst controlling for baseline levels in the proposed mechanism or outcome within the befriending group only (Study 2). Partial rank correlations were calculated (Conover, 1999). Estimates for the confidence intervals for the r-values were calculated using this online calculator here: <http://vassarstats.net/rho.html>. The size of the associations (r-values) were interpreted using the conventions outlined by Hattie, J. (2011) [0.00-0.10 = no effect, 0.10-0.20= small effect, 0.20-0.30 = moderate effect, r > 0.30 = large effect] (Hattie, 2011).

1. **Logic Diagram for Study 2**

**
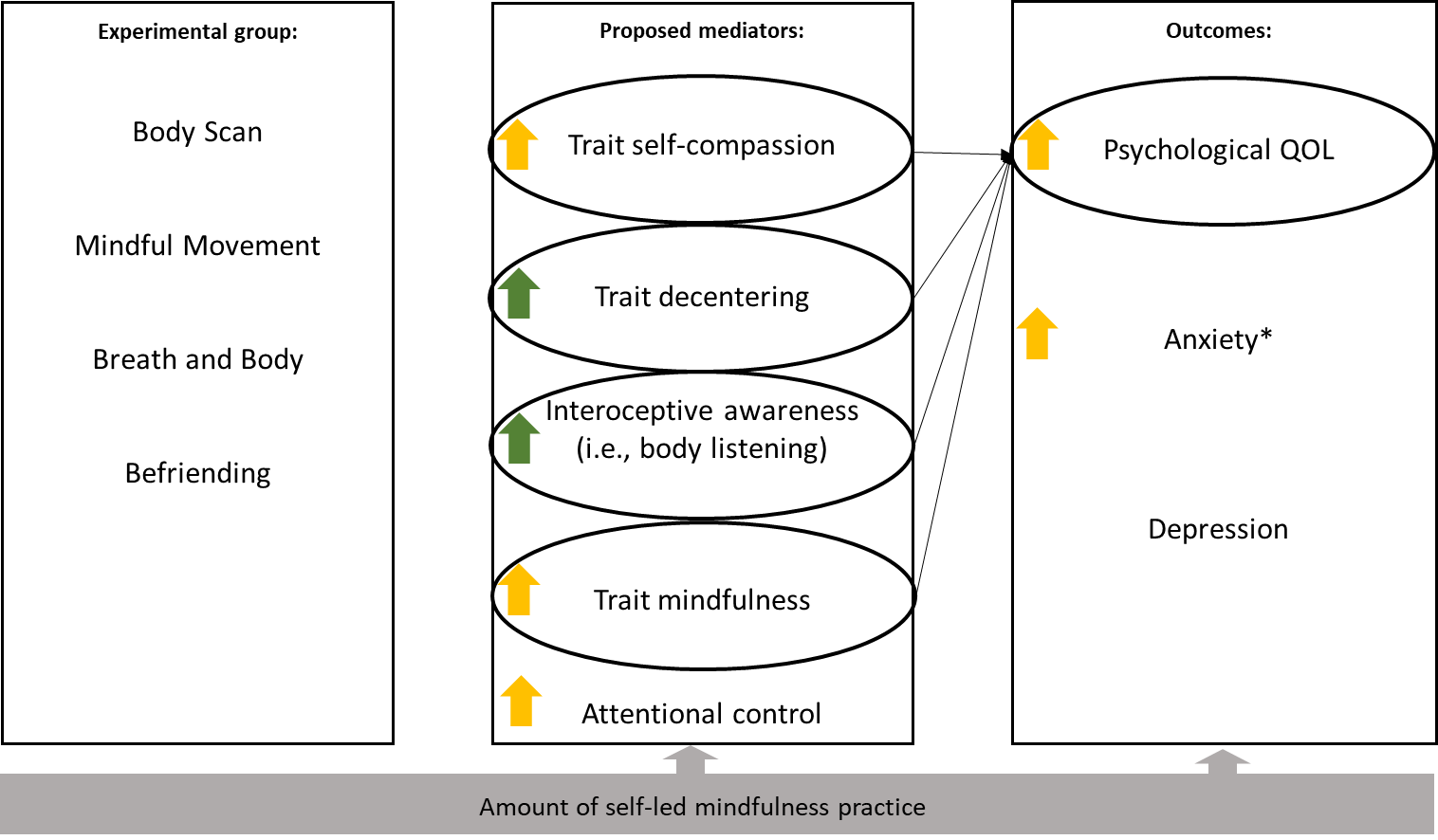
**

This logic diagram illustrates the key hypotheses that were generated from Study 2. The large arrows are used to indicate the within-group effects within the whole sample for the candidate mechanisms and outcomes (e.g., increase in trait self-compassion and psychological quality of life). The yellow arrows are used to indicate small to medium effects whereas the green arrows are used to indicate medium to large effects. *Although, a slight increase in anxiety was found within the whole sample, it is hypothesized that this is due to a possible floor effect and therefore this effect was removed from the logic diagram. No pre-post effect was found for depression. The solid black lines with arrows indicate the small to large correlations found within the whole sample between changes in the candidate mechanisms and the amount of self-reported mindfulness practice and changes in outcomes. The aim of this diagram is to help guide future research. Please note that analyses within the whole sample were prioritized due to larger sample size and not all correlations are reported.

1. **Additional information**

**The association between pre-post scores for state and trait decentering and mindfulness**

| **Variable** | **Time point** | **Output** |
| --- | --- | --- |
| Trait and state mindfulness (pre) | T1, T2 | *r* _(120)_ = 0.30 [0.13, 0.45], *p* = 0.001 |
| Trait and state mindfulness (post) | T4 | *r* _(117)_ = 0.25 [0.07, 0.41], *p* = 0.008 |
| Trait and state mindfulness (pre-post differential scores) | T1-T4, T2-T4 | *r* _(116)_ = 0.39 [0.23, 0.53], *p* < 0.001 |
| Trait and state decentering (pre) | T1, T2 | *r* _(120)_ = 0.54 [0.40, 0.66], *p* < 0.001 |
| Trait and state decentering (post) | T4 | *r* _(117)_ = 0.55 [0.41, 0.66], *p* < 0.001 |
| Trait and state decentering (pre-post differential scores) | T1-T4, T2-T4 | *r* _(116)_ = 0.43 [0.27, 0.57], *p* < 0.001 |

This table depicts Spearman’s rho correlations between pre- and post-scores and differential scores for state and trait mindfulness and decentering using a complete-case approach within the whole sample.

1. **References for Supplementary Material**

Conover, W. J. (1999). *Practical Nonparametric Statistics* (3rd ed.). Wiley.

Hattie, J. (2011). *Visible learning for teachers: Maximise impact on learning* (1st ed.). <https://doi.org/10.4324/9780203181522>

Kline, R. (2004). *Beyond significance testing: Reforming data analysis methods in behavioral research.* (1st ed.). American Psychological Association.

Kroenke, K., Spitzer, R. L., & Williams, J. B. W. (2001). The PHQ-9. *Journal of General Internal Medicine*, *16*(9), 606–613. https://doi.org/10.1046/j.1525-1497.2001.016009606.x

Montero-Marin, J., Taylor, L., Crane, C., Greenberg, M. T., Ford, T. J., Williams, J. M. G., García-Campayo, J., Sonley, A., Lord, L., Dalgleish, T., Blakemore, S.-J., Team, M., & Kuyken, W. (2021). Teachers “finding peace in a frantic world”: An experimental study of self-taught and instructor-led mindfulness program formats on acceptability, effectiveness, and mechanisms. *Journal of Educational Psychology*, *113*(8), 1689. https://doi.org/10.1037/edu0000542

Segal, Z., Williams, M., & Teasdale, J. (2018). *Mindfulness-Based Cognitive Therapy for Depression, Second Edition*. Guilford Publications.

Silva, P. A. B., Soares, S. M., Santos, J. F. G., & Silva, L. B. (2014). Cut-off point for WHOQOL-bref as a measure of quality of life of older adults. *Revista de Saúde Pública*, *48*, 390–397. https://doi.org/10.1590/S0034-8910.2014048004912

Spitzer, R. L., Kroenke, K., Williams, J., B. W., & Lowe, B. (2006). *A Brief Measure for Assessing Generalized Anxiety Disorder: The GAD-7*. https://doi.org/doi:10.1001/archinte.166.10.1092

Strauss, C., Gu, J., Montero-Marin, J., Whittington, A., Chapman, C., & Kuyken, W. (2021). Reducing stress and promoting well-being in healthcare workers using mindfulness-based cognitive therapy for life. *International Journal of Clinical and Health Psychology*, *21*(2). https://doi.org/10.1016/j.ijchp.2021.100227

Thompson, B. L., & Waltz, J. (2007). Everyday mindfulness and mindfulness meditation: Overlapping constructs or not? *Personality and Individual Differences*, *43*(7), 1875–1885. https://doi.org/10.1016/j.paid.2007.06.017

Williams, J. M. G., Baer, R., Batchelor, M., Crane, R. S., Cullen, C., De Wilde, K., Fennell, M. J. V., Kantor, L., Kirby, J., Ma, S. H., Medlicott, E., Gerber, B., Johnson, M., Ong, E.-L., Peacock, J. W., Penman, D., Phee, A., Radley, L., Watkin, M., & Taylor, L. (2022). What next after MBSR/MBCT? An open trial of an 8-week follow-on program exploring mindfulness of feeling tone (vedanā). *Mindfulness*, *13*(8), 1931–1944. https://doi.org/10.1007/s12671-022-01929-0

Williams, M., & Penman, D. (2011). *Mindfulness: A practical guide to finding peace in a frantic world*. Piatkus.
